# Effect of functional oils or probiotics on performance and microbiota profile of newly weaned piglets

**DOI:** 10.1038/s41598-021-98549-w

**Published:** 2021-09-30

**Authors:** Alexandre Maciel Vieira, Amanda Pires Sessin, Tatiany Aparecida Teixeira Soratto, Paula Gabriela da Silva Pires, Kátia Maria Cardinal, Glauber Wagner, Lucélia Hauptli, André Luis Ferreira Lima, Fabiano Dahlke, Diego Peres Netto, Priscila de Oliveira Moraes

**Affiliations:** 1 Department of Animal Science and Rural Development, Universidade Federal de Santa Catarina, Florianópolis, Santa Catarina Brazil; 2 Laboratory of Bioinformatics, Center of Biological Sciences, Universidade Federal de Santa Catarina, Florianópolis, Santa Catarina Brazil; 3 Laboratory of Animal Science, Department of Animal Science, Universidade Federal do Rio Grande do Sul, Porto Alegre, Rio Grande do Sul Brazil

**Keywords:** Microbiology, Microbial communities

## Abstract

The study aimed to evaluate a commercial blend of functional oils based on liquid from the cashew nutshell and castor oil as a growth promoter in newly weaned piglets. A total of 225 piglets, castrated males and females with 28 days of age were randomly distributed in pens with 15 animals composing three treatments and five repetitions. The treatments were: control (without the inclusion of additives), probiotics, or functional oils. The performance was evaluated. At 50 days of age, a pool of fresh feces from 3 animals/repetition was collected to perform the sequencing of microbiota using the Illumina MiSeq platform. Supplementation with functional oils improved the piglets' daily weight gain and feed conversion ratio (P < 0.05) in the first weeks of the experiment, which resulted in higher final live weight (P < 0.05) in the phase when compared to the control treatment (24.34 kg and 21.55 kg, respectively). The animals that received probiotics showed an intermediate performance (23.66 kg final live weight) at the end of the 38 experimental days. Both additives were effective in increasing groups essential for intestinal health, such as *Ruminococcaceae* and *Lachnospiraceae*. The functional oils were more effective in reducing pathogenic bacteria, such as *Campylobacter* and *Escherichia coli*. In conclusion, the use of functional oils optimized performance and effectively modulated the microbiota of newly weaned piglets.

## Introduction

Several stressors occur during the weaning of piglets, such as separation from the mother and siblings, transport and handling, or reformulation of the social hierarchy due to the mixing of different groups of piglets. These stressors lead to oxidative stress, as well as to inflammation and dysbiosis, which, may consequently result in diarrhea, decreased growth and increased mortality rate^1^. Diarrhea is an important factor with negative economic impact in the nursery, and its main pathogenic agent is enterotoxigenic *Escherichia coli* (ETEC) K88^2^.

For decades, antibiotics have been fed at low dosages to nursery pigs to minimize the negative impact of weaning^3^. However, most countries have been implementing policies and regulations to reduce or ban the use of antimicrobials in animal production. These changes are motivated by the overuse of antibiotics, which results in the appearance of super resistant bacteria, compromising their effectiveness in human and/or animal health^4^.

The regulatory restrictions in the use of antibiotics coupled with the reduced number of compounds with similar productive potential has resulted in reductions in performance and higher pig mortalities, especially during the nursery phase^5^. Therefore, new compounds are needed to replace the antibiotics in the diet of weaned piglets.

Probiotics are live microorganisms that modulate the host's intestinal microbiota. Their beneficial effects are associated with their adherence to the epithelium, the inhibition of the growth and the reduction of toxins produced by pathogenic bacteria^6^. For example, supplementation with *Lactobacillus* spp. has been shown to reduce the fecal counts of *Salmonella serovar Typhimurium* KCTC 2515 and *Escherichia coli* KCTC 2571 in weaned piglets, increasing the average daily gain and average daily feed intake^7^.

Functional oils are defined as oils that have an action beyond their nutritional value^8^, and are being disseminated in the pig industry due to their antimicrobial action and the modulation of intestinal microbiota^1^.

The liquid from the cashew nut shell is a renewable resource, with cardol in its composition, which has an antimicrobial potential, mainly against gram-positive bacteria such as *Streptococcus mutans*, *Bacillus subtilis* and *Staphylococcus aureus*^9^. Additionally, ricinoleic acid, the main component of castor oil, acts by denaturing and coagulating proteins in bacterial cell wall^10^.

The commercial mixture containing functional oils from cashew nut liquid and castor oil has already demonstrated positive effects on performance, modulation of intestinal microbiota and immune system of broilers challenged by coccidiosis^11–14^. However, there are no published studies analyzing the effects of this product on the performance and intestinal microbiota of swine.

Our hypothesis is that the use of functional oils or probiotics will provide piglets with better performance and an intestinal microbiota beneficial to the animal due to the modulation caused by the additives. Thus, the objective of this study was to evaluate the effects of supplementing a probiotic or a commercial blend of cashew nutshell liquid and castor oil, on the performance, blood parameters and intestinal microbial composition of weaned piglets.

## Results

### Performance and frequency of diarrhea

The effects of additives on the performance are shown in Table 1. The DWG in phases 1, 2 and the general average was higher for the group receiving the blend of functional oils in the diet when compared to the control group, the probiotic group showed an intermediate result (P < 0.05). Similar results were observed for the live weights at 57 and 66 days of life. Pigs supplemented with the blend of functional oils ate less than the other groups (p < 0.05), which also resulted in a better average FCR (p < 0.05).

**Table 1:** Effect of different feed additives on the performance of weaned pigs in phase 1 (28–43 days old), phase 2 (43–57 days old), phase 3 (57–66 days old), and on average (28–66 days old).

Item	Additives				p-value
	Control^1^	Oils^2^	Probiotic^3^	SEM^4^	
**Live weight, kg**					
Day 28	8.66	8.77	8.83	0.3261	0.6897
Day 43	11.25	12.21	11.5	0.4370	0.2936
Day 57	17.90^b^	19.84^a^	18.68^ab^	0.6206	0.0427
Day 66	21.55^b^	24.34^a^	23.66^ab^	0.8046	0.0498
**Average daily gain, kg**					
Phase 1	0.182^b^	0.228^a^	0.211^ab^	0.0172	0.0147
Phase 2	0.522^b^	0.662^a^	0.587^ab^	0.0181	0.0031
Phase 3	0.431	0,491	0.494	0.0265	0.2845
Average	0.313 ^b^	0.429^a^	0.395^ab^	0.0201	0.0165
**Daily feed intake, kg**					
Phase 1	0.325	0.328	0.320	0.0100	0.2026
Phase 2	0.879	0.770	0.814	0.0480	0.3368
Phase 3	0.943	0.986	1.005	0.0320	0.3564
Average	0.725^a^	0.688^b^	0.709^a^	0.0210	0.0350
**Feed conversion ratio, kg kg**					
Phase 1	1.771^a^	1.482^b^	1.538^ab^	0.2925	0.0371
Phase 2	1.726^a^	1.350^b^	1.387^ab^	0.0512	0.0385
Phase 3	2.174	1.932	2.050	0.3401	0.1689
Average	2.275^a^	1.732^b^	1.853^a^	0.0951	0.0470

Least squares: based on observations of 5 stalls per diet.

^1^Control: without the inclusion of zootechnical additives; ^2^Probiotics: inclusion of 0.4% probiotic. Probiotic composition: *Bacillus subtilis*, *Enterococcus faecium*, *Lactobacillus acidophilus*, *Bifidobacterium bifidum*, *Saccharomyces cerevisiae*; ^3^Functional oils with inclusion of 0.2% Essential + 0.15% Integrity; ^4^SEM: standard error of the mean.

^ab^Averages within the same row with different overwrites are statistically different (P < 0.05).

**Figure 1:**
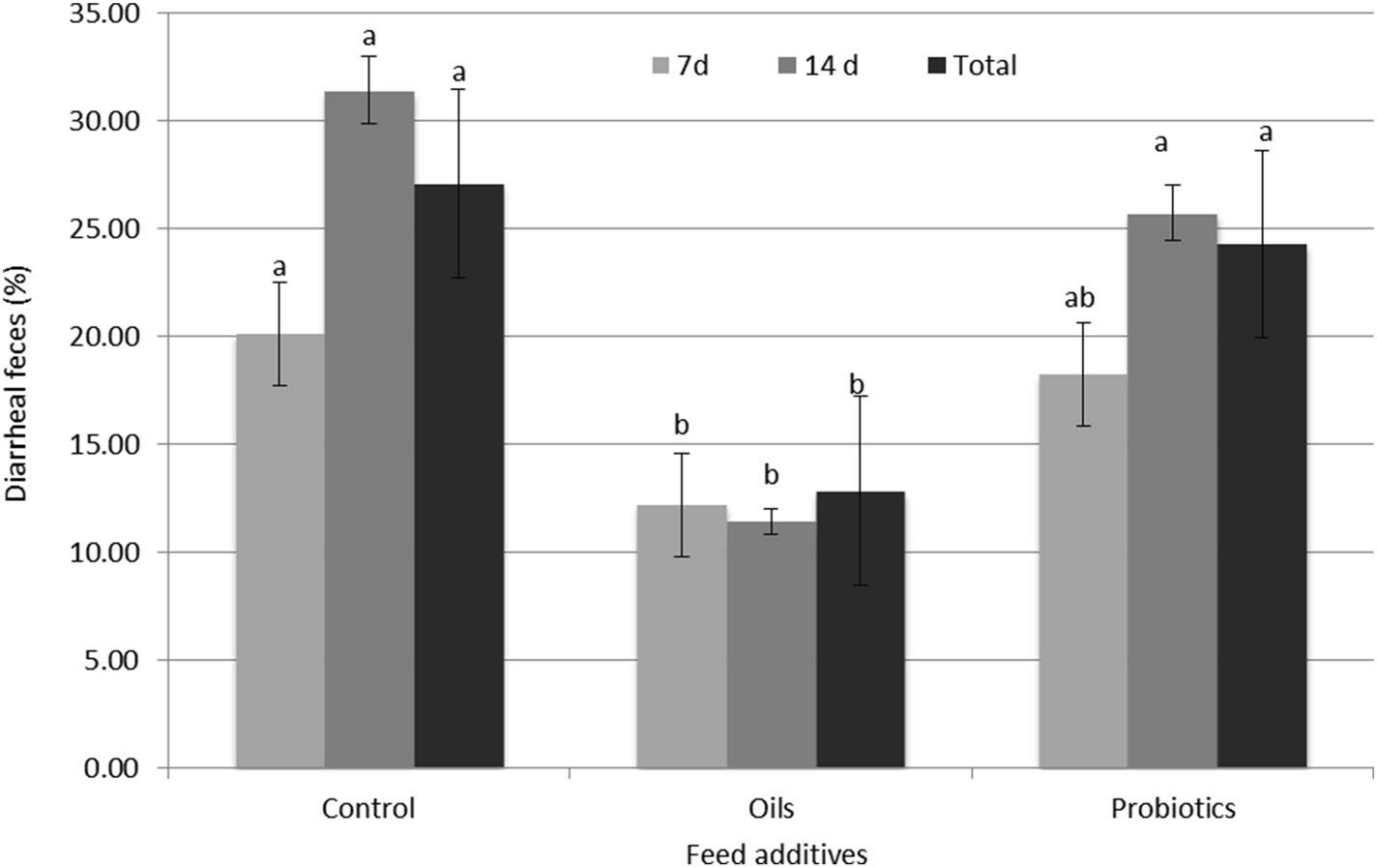
Frequency of diarrheal feces over 2 weeks and in the total period of rearing piglets submitted to different experimental diets in the nursery phase. Control: without the inclusion of zootechnical additives; oils: functional oils with inclusion of 0.2% Essential + 0.15% Integrity; ^2^Probiotics: inclusion of 0.4% probiotic. Probiotic composition: *Bacillus subtilis*, *Enterococcus faecium*, *Lactobacillus acidophilus*, *Bifidobacterium bifidum*, *Saccharomyces cerevisiae*; ^ab^Averages within the same row with different overwrites are statistically different (P < 0.05).

The percentage of diarrheal feces was lower for the pigs that received the functional oils (p < 0.05) (Fig. 1).

### Intestinal microbiota and leukogram

The study sequenced a total of 15 fecal pool samples, being three treatments (control, oils, and probiotics) with 5 replicates collected at 50 days of trial. Illumina sequencing analysis of the V3-V4 region of the 16S rDNA gene of 15 fecal pool samples generated a total of 967,431 trimmed quality sequences with an average number of reads per sample of 64,495.4 ± 18,795.6 (Table S1). One sample (181113520761-1-1-1) of the functional oils treatment was disregarded due to the low number of sequences compared to the other samples. The reads were processed and classified into 1690 amplicon sequence variants (ASVs).

The rarefaction curves generated from ASVs (Fig. S1) showed high sequencing coverage in all samples. The rarefaction curves tended to reach the saturation plateau, this result demonstrates that the microbiota of the 14 samples was deep enough to estimate the phenotypic richness and the diversity of the microbial community.

### Variation in alpha and beta diversity of the microbiota.

#### Alpha diversity

**Figure 2:**
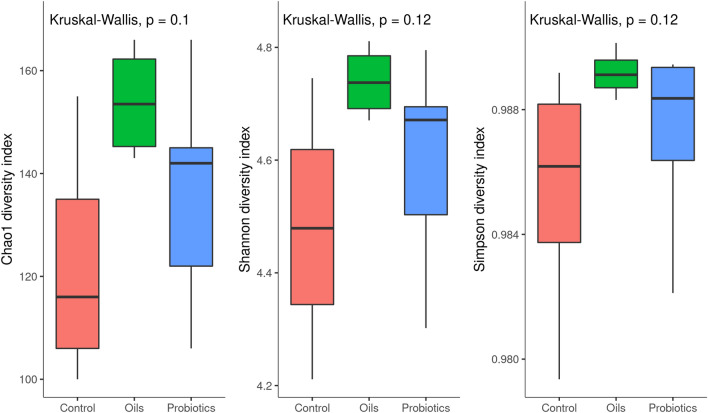
Alpha diversity of fecal pool samples of weaned piglets at 50 days of trial. The study consisted of three feed additives in the nursery phase: basal diet (control), Functional Oils or Probiotics.

The Chao 1 index was based on the richness amplicon sequence variant (ASVs) present in the sample. The Shannon index considered uniformity in taxa abundance, and the Simpson index was based on the taxa abundance dominance (Fig. 2). The Chao, Shannon and Simpson indices showed no significant difference among the three treatments (p > 0.05). However, there is a tendency to increase the indices and the uniformity in the functional oils group followed by the probiotics, in comparison to control.

#### Beta diversity

**Figure 3:**
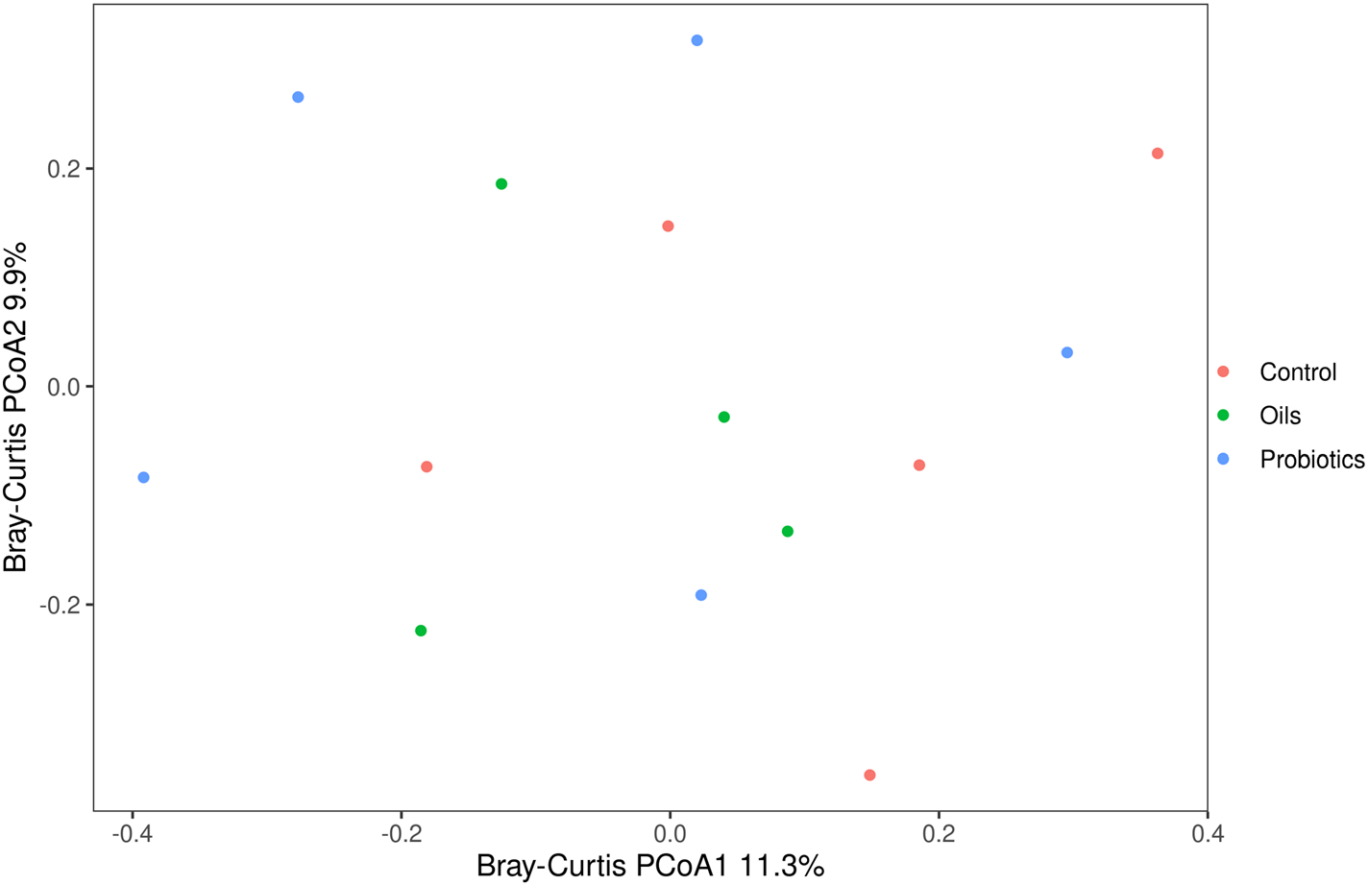
Principal coordinate analysis (PCoA) of beta diversity based on Bray–Curtis dissimilarity of fecal pool samples of weaned piglets at 50 days of trial. The study consisted of three feed additives in the nursery phase: basal diet (control), Functional Oils or Probiotics. Comparison among Oils, Probiotics and Control treatments (Adonis with 999 permutations, p = 0.337).

The Bray–Curtis dissimilarity index (BC) was used to analyze inter-individual differences. Based on the PCoA graph (Fig. 3), it was possible to verify that the microbial populations of the animals of the three treatments showed homogeneous dispersion (p > 0.05). The index showed that the treatments had a similar microbial composition (Adonis with 999 permutations, p = 0.337).

**Figure 4:**
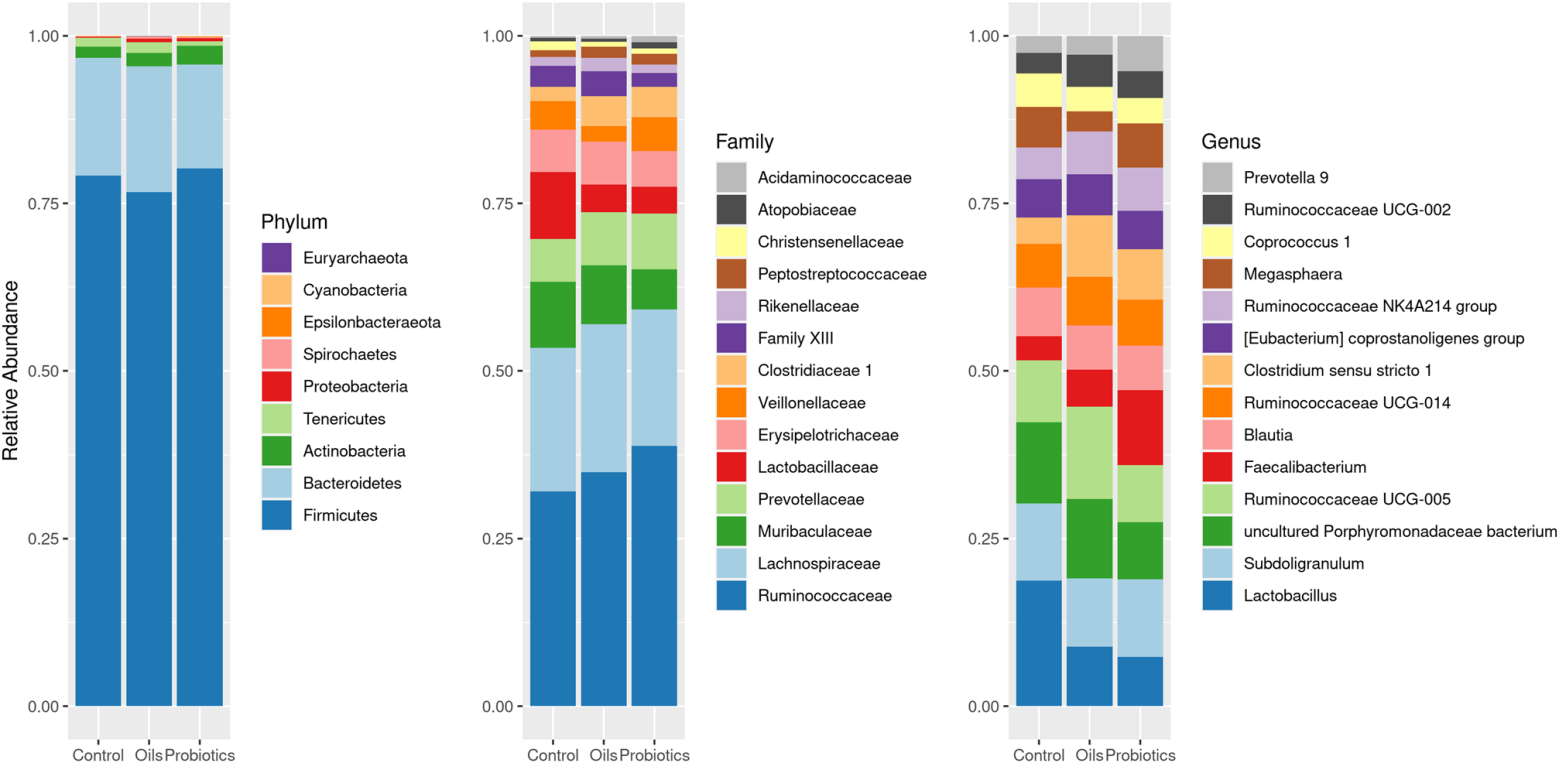
The relative abundance of the fecal microbiota of weaned piglets at 50 day of age receiving three treatments: basal diet (Control), Functional oils (Oils) or Probiotics. The 14 most abundant taxa at Family and Genus level.

All sequences were classified into nine phyla, although four phyla were more common (> 1%): *Firmicutes, Bacteroidetes, Actinobacteria* and *Tenericutes. Firmicutes* was the most abundant phylum in all treatments (> 76%) (Fig. 4). A complete list of the identified sequences (relative abundance) per treatment is provided in Supplementary Table 3. *Bacteroidetes* and *Tenericutes* were more abundant for the blend and less abundant for probiotics treatment. *Actinobacteria* was more abundant in the blend of functional oils and in the group supplemented with probiotics when compared to the control group.

Thirty-five families (35) were identified, of which fourteen (14) had relative abundance > 1% (Table S2 and Fig. 4). The *Ruminococcaceae* family was predominant in all groups (> 30%) and showed an increase in animals supplemented with probiotics with a log2 FoldChange of 0.6266 (padj < 0.001) (Fig. 4). The *Lachnospiraceae* family was the second most abundant (> 19%). Compared to the control, *Lactobacillaceae* were less abundant for both additives (~ 9% vs. ~ 3%). Similar behavior was observed for *Muribaculaceae*. *Clostridiaceae* 1 was more abundant for both additives.

Piglets fed the control diet showed a higher concentration of *E.coli* when compared to the treatment with the blend of functional oils, whereas the treatment with probiotic was not different from any of the other two treatments (P < 0.05; Fig. S2).

The leukogram (Table 2) showed that the concentration of lymphocytes in the control and probiotic groups was higher when compared to treatment with functional oils (p < 0.05). The other parameters, leukocytes, neutrophils, eosinophils and monocytes did not differ statistically among treatments (P > 0.05). Mortality tended to be lower in pigs supplemented with either of the two additives.

**Table 2:** Blood analysis of piglets at 50 days of age receiving different feed additives.

Item	Additives				p-value
	Control^1^	Oils^2^	Probiotic^3^	SEM	
Mortality %	10.00	2.65	4.85	2.228	0.0504
Leukocytes, mm^3^	21,080	17,060	20,020	0.485	0.5218
Neutrophils, mm^3^	13,297	11,740	13,003	0.558	0.8490
Lymphocytes, mm^3^	6046^a^	3363^b^	5418^a^	0.437	0.0056
Eosinophils, mm^3^	926	606	649	1.271	0.7989
Monocytes, mm^3^	810	1262	948	0.755	0.2979

Least squares means based on 5 pen observations per diet.

^1^Control: without the inclusion of zootechnical additives; ^2^Probiotics: inclusion of 0.6% probiotic. Probiotic composition: *Bacillus subtilis*, *Enterococcus faecium*, *Lactobacillus acidophilus*, *Bifidobacterium bifidum*, *Saccharomyces cerevisiae*; ^3^Blend of functional oils with the inclusion of 0.2% Essential + 0.15% Integrity.

^ab^Averages within the same line with different overwrites are statistically different (P < 0.05).

## Discussion

The first week after weaning is the most the critical phase of weaning, when the diet changes from highly digestible (breast milk) to a more complex digestible solid food. This change directly affects the physiology of the piglets' gastrointestinal tract that is not fully adapted^15^, causing intestinal and immune system dysfunction and result in less health, growth, and feed intake^16^. The use of functional oils resulted in greater average daily weight gain (DWG) and feed conversion (FCR) in piglets during the first and second periods, which also resulted in heavier pigs at the end of the experiment, when compared to the control treatment.

Although it is not clear how the supplementation of functional oils improved pig growth, several mechanisms have been proposed, including antimicrobial activity, such as reducing pathogenic stress or increasing the abundance of beneficial microorganisms in the intestine, such as *Lactobacillus * spp.^1^; protecting intestinal villi and regulating enzyme activity^17^; also, modulating the intestinal microbiota and increasing the absorption of nutrients^18^.

Previous studies have shown that the supplementation of a blend of essentials oils (cinnamaldehyde and thymol) in the diet of weaned piglets positively influenced characteristics of zootechnical interest, such as higher DWG and lower FCR (P < 0.05)^19,20^, similarly to the results of the present study. Evaluating the supplementation of *L. acidophilus* in weaned piglets^21^, observed an improvement in DWG and FCR (P < 0.05) compared to the control group, and the same result was observed in the group receiving probiotics in the present study.

Probiotics act by modulating the microbiota, mainly by adhesion and competitive exclusion of pathogens at binding sites in the intestinal epithelium^6^. The blend of functional oils used in this study acts by modulating the immune system and the intestinal microbiota with antimicrobial action, mainly against gram-positive bacteria^11,13^. Both additives provided better performance and modulation of the microbiota in the face of the weaning challenge due to different mechanisms of action. In this study, there was no difference in microbial diversity between the additives, estimated by the Chao, Shannon and Simpson indices. Similar results were found by^22^ and^23^, who evaluated the supplementation of essential oils and probiotics in weaned piglets, respectively. Although without a statistical difference, there was a tendency to increased diversity for the group supplemented with functional oils. The increase in microbial richness and diversity can be seen as a predictor of the stability of the microbial ecosystem^24^.

It is important to highlight that for the microbiome analysis, the low number of replicates and the collection were conducted in just one period. The collection was performed at 50 days of age (22 days after weaning). The piglets' microbiota is more stabilized at this age^6^. However, evaluating the role of additives in the intestinal environment, even in the period when the microbiota is most stabilized, is extremely important to understand their role in animal performance. In this way, relative abundance was discussed as an exploratory analysis.

The phyla *Firmicutes, Bacteroidetes, Proteobacteria* and *Actinobacteria*, in decreasing order concerning relative abundance, are predominant in swine gastrointestinal tracts^6^. These results are in line with the findings in this study.

The ratio presented between *Firmicutes/Bacteroidetes* was 4.50, 5.51 and 5.17 respectively for the control treatments, functional oils and probiotics. It has been shown that heavier pigs tend to have a higher *Firmicutes* vs. *Bacteroidetes* ratio than lighter animals^25^. In this study there was no statistical difference, only a greater numerical relationship for the treatments with functional oils and probiotics. Functional oils modify the composition of intestinal microbiota, increasing the relative abundance of *Firmicutes* in the intestine, as demonstrated in broilers in vivo studies by^26^ and in pigs by^22^. It is necessary to highlight that the increase in the *Firmicutes*. *Bacteroidetes* ratio is a natural trend found in the healthy intestinal microbiota of matured piglets. However, in dysbiotic situations, such as those caused by weaning, it can result in a decrease in *Firmicutes* and an increase in *Bacteroidetes*. These changes provide a favorable environment for the proliferation of some pathogenic genera of this second phylum and, consequently, a reduction in the feeding efficiency of the animals^27^.

Also, a large reduction in *Bacteroidetes* can cause damage to the host. Bacteroidetes, despite encompassing some pathogenic species, are known to have a large number of genes that encode active carbohydrate enzymes and can readily switch between different energy sources, in addition to being an important source of propionate^28,29^. Additionally, *Firmicutes* has members nutritionally more specialized in the degradation of complex substrates, such as plant cell walls, starch particles and mucin^30^. Therefore, a stable relationship between *Firmicutes* and *Bacteroidetes* can result in better utilization of the diet by animals. In the present study, both additives were effective in maintaining the *Firmicutes. Bacteroidetes* ratio, a fact that may have contributed to the better performance of the animals in both supplemented groups.

*Tenericutes* also seem to be involved in improving the use of nutrients by the host. In a study with piglets^31^, found a positive correlation of this phylum with a better apparent digestibility of crude fiber. However, data on the relationship of this phylum with animal performance are still scarce. Proteobacteria are known to harbor numerous opportunistic pathogens in animals and humans, including *Escherichia coli*, *Escherichia Shigella*, *Salmonella*, *Brucella*, *Rickettsiaos* spp. Thus, it is associated with several intestinal disorders and infectious diseases^32,33^. In this study, the use of functional oils increased *Tenericutes* and inhibited Proteobacteria.

Although only the Ruminococcaceae family showed different relative abundance between treatments. The supplementation with both additives kept the relative abundance of *Lachnospiraceae* stable, increased the *Ruminococcaceae* and *Prevotellaceae* abundances, and reduced *Lactobacillaceae.* These four families are known to be part of a group fundamental to the microbial activity in the piglets' intestines^34^.

*Ruminococcaceae*, for example, are associated with fiber degradation and higher concentrations of butyrate in piglets^35^. Butyrate contributes to a better absorption of nutrients stimulating the growth of intestinal mucosa cells, improving the retention of calcium and phosphorus in the diet, mitigating the challenge of weaning^36^ and inducing secretion of mucin, a glycoprotein, which forms a protective layer in enterocytes^37^. The genus *Faecalibacterium*, from *Ruminococcaceae*, has been negatively associated with feed efficiency in pigs. In the present study, this genus was present in a higher percentage in the probiotics group when compared to the other treatments.

*Prevotella* and *Lachnospiraceae* are positively correlated with gene functions associated with the metabolism of amino acids, energy, cofactors and vitamins, indispensable to the host^38^. *Prevotella* has also been positively associated with higher luminous IgA concentrations and body weight in weaned piglets, showing its importance to the health of piglets^38^.

Lactobacillus is prevalent in the fecal microbiota of piglets in early life and tends to decrease during the weaning transition^39^. Several species of Lactobacillus are associated with beneficial characteristics for the host. It has been shown that the swine microbial population differs between more efficient and less efficient animals. More efficient animals have a higher number of *Lactobacillus *spp. than less efficient animals^40^.

Interestingly, the opposite behavior was observed in the present study, where the blend and probiotics groups showed numerically a less relative abundance of this genus (~ 3%), but better performance, while the control group, greater relative abundance (~ 9%) and less performance. The higher concentration of Lactobacillus in the control group may be associated with the activation of the immune system of animals in this group in the face of a greater challenge (as evidenced by the higher rate of diarrheal incidence and *E. coli* count in feces of these animals). Many species of Lactobacilli act in the innate and acquired system stimulating immune cells to release pro-inflammatory cytokines, such as tumor necrosis factor alpha (TNF-α), gamma interferon (IFN-γ) and interleukin-12 (IL-12)^41^. It is possible to conclude that the lower concentration of Lactobacillus in the functional oils and probiotics groups did not result in losses to the animals' performance.

The *Clostridiaceae* family is known to have different species, including *C. pectinovor one*, *Clostridium butyricum*, *Clostridium perfringens*. *Clostridium butyricum*, for example, acts in the production of short-chain fatty acids and has been studied as a probiotic in other animal species, such as broilers, where resulted in the improvement of the function of the intestinal barrier and the inhibition of pathogens^42^. The groups supplemented with Functional Oils and Probiotics presented an average of 4% and the control 2% of relative abundance of this family.

Curiously, the supplementation with probiotics (*Lactobacillus* spp., *Bifidobacterium* and *Saccharomyces cerevisiae*) did not result in an increase of these genera, other than for *Bifidobacterium*, in the fecal microbiota of piglets. Two factors may explain this result. On one hand, the technology and conditions involved in the preservation of these probiotics, which can negatively influence the viability of the strains used until they reach the small intestine of piglets. On the other hand, it is known that different species of bacteria are subject to adverse conditions in the gastrointestinal tract, and when a exogenous microorganism is fed as a probiotic and enters the gastrointestinal tract, it needs to compete with the existing microbiota ecosystem^43^. Thus, in some cases, unfavorable circumstances may end up hindering the proliferation of the microorganism used as a probiotic^44^.

The blend of functional oils kept the *Muribaculaceae* family (phylum *Bacteroidetes*) stable when compared to the other treatments. Bacteria in this group have been positively related to the regulation of genes for carbohydrate metabolism in mice^45^. *Muribaculaceae* members may be involved in the fermentation of starch into propionate, and its composition is an important predictor of higher concentrations of short-chain fatty acids in healthy intestinal microbiota of the animals^46^.

In contrast, the Probiotic provided a greater relative abundance of *Veillonellacea*e (4%) compared to the functional oils group (2%), but not compared to the control (5%). This family is directly involved in metabolic functions related to proteins and enzymes essential to the host^47^.

*Shigella* spp. and *Escherichia coli* are closely related and, although they have some differences, they are considered unique genome species. *Shigella* spp. are among the most important enteric pathogens that cause bacillary dysentery worldwide, especially in humans^48^. As observed in *Enterobacteriaceae*, it was very low, 0.07% for the control, 0.03% and 0.04% for the functional and probiotic oils. This may have occurred due to the age of sample collection or due to the limitation of the technique. In this same sense, using the *E. coli* culture technique, it significantly reduced (P < 0.05) *E. coli* in the feces of piglets treated with the blend, a result that agrees with those observed by^22^. These authors showed that supplementation of 100 ppm of functional oils based on thymol and cinnamaldehyde to the control diet reduced the *E. coli* count in the feces of weaned piglets.

The reason for these effects may be associated with the antimicrobial activity of phytogenics, demonstrated in vitro by^42^. The authors evaluated a mixture based on thymol and cinnamaldehyde and observed its ability to damage the cell membrane and alter the morphology of *E. coli* and *S. aureus* pathogenic cells. Similarly^49^, evaluated the supplementation of six essential oils in vivo (including thymol, carvacrol, and eugenol) in piglets challenged by enteropathogenic species (*E. coli*, *Salmonella* spp. and *C. perfringens*), reporting antimicrobial activity of these essential oils against at least one of these species. The intermediate results of the Probiotic group agree with other studies^21,44^, which also reported the ability of the Probiotic supplementation (*L. acidophilus*, *Pediococcus acidilactici*) to reduce fecal *E. coli* in weaned piglets.

The genus Campylobacter was found in 0.03% of the microbiota when supplemented with functional oils and 0.05% and 0.08%, respectively for the control and probiotic group. *Campylobacter* is the predominant bacterial agent in diarrheal piglets, reducing the relative abundance of bacterial species of the classes *Bacteroidia* and *Clostridia*. Both ferment the non-digestible carbohydrate. This reduction results in less production of short-chain fatty acids, which are the main metabolites of the intestinal microbiota, and which could promote barrier function and maintain a healthy and slightly acidic environment in the colon^42^.

The erythrocyte, hematocrit, hemoglobin and platelet values of all treatments varied within the reference intervals for young piglets, as recommended by^50^. This indicates that the animals were, in general, in good health and not anemic. Similarly^47^, found no significant effect of supplementing 40 ppm of functional Oregano oils on the hematological status of weaned piglets.

The difference in leukocytes circulating in pigs may be associated with two specific factors: inflammatory state or stress state, caused during the weaning period^51^. In a study with rats under stress, the levels of circulating inflammatory leukocytes increased by directly stimulating the proliferation of hematopoietic stem cells^52^. Similarly, it has been reported that some types of stressors have increased the total leukocyte count and the proliferation of T cells in pigs^53^.

In another study, evaluating hematological parameters in piglets challenged by Salmonella^54^, found no significant differences in Salmonella concentrations in the animals' feces, and suggested that variations in hematological parameters, in that study, were more related to the state of stress than to actual infection by this pathogen. On the other hand, the increase in the percentage of lymphocytes in the blood in piglets challenged by enterotoxigenic *Escherichia coli*^17^ was associated with a change in the inflammatory state of these animals, due to the challenge. Opposite results weres observed in this study for the blend of functional oil group, suggesting that it can mitigate *E. coli* infection.

Research that reports the impact of additives during the microbiota transition period, that is, before weaning and in the first 14 days of weaning are still needed to elucidate the effect of additives on piglet intestinal health.

The commercial blend of functional oils based on cashew nut shell liquid and castor oil improved the performance of piglets weaned during the nursery period. The animals that received probiotics presented intermediate performance and the piglets that did not receive either additive performed worse. The use of functional oils reduced the concentration of *Escherichia coli* in piglet feces at 50 days of age, demonstrating a modulating effect on the intestinal microbiota of newly weaned piglets.

## Material and methods

The described study was performed according to protocol nº 3665110718 and approved by the Ethics Committee on the Use of Animals at the Universidade Federal de Santa Catarina, performed in strict accordance with the NIH Guide for the Care and Use of Laboratory Animals, and reported according to the ARRIVE guidelines (https://www.nc3rs.org.uk/arrive-guidelines). This experiment was performed in a group of piglets that were reared in a commercial pig farm, located in the municipality of Jaguaruna—Santa Catarina/Brazil.

### Animals, facilities and diets

A total of 225 piglets descended from commercial lines of F1 females (Landrace × Large White) with tricross males (Hampshire × Duroc × Pietrain), weaned at 28 days of age, females and castrated males, weighing 8.54 ± 0.622 kg were randomly distributed in 15 pens, with a density of 0.3 piglets/m^3^, hollow wooden floor and equipped with automatic feeder and drinker.

Water and feed were offered ad libitum throughout the experimental period. The study was divided into the three phases according to their age. Phase I: from 28 to 43 days; phase II: from 43 to 57 days; and phase III: from 57 to 66 days. All diets were formulated to meet the nutritional requirements of piglets^55^. The only difference among diets was the additive used, as described in Supplementary Table 3. The treatments were: (1) control group—without the inclusion of zootechnical additives; (2) probiotic group—inclusion of 0.6%; (3) blend group of functional oils with inclusion of 0.35% (0.2% of Essential + 0.15% of Integrity). All feed additives were included in the diets by replacing inert (kaolin) in the basal diet in all phases. Additive doses were used according to the manufacturer's recommendation. The commercial product Integrity is basically composed of cardanol (75 g/kg) and cardol (15 g/kg) and Essential by cardanol (200 g/kg), ricinoleic acid (90 g/kg) and cardol (40 g/kg). The composition of the probiotic used was *Bacillus subtilis (*3.66 × 10^7^ cfu/kg*), Enterococcus faecium* (3.5 × 10^6^ cfu/kg)*, Lactobacillus acidophilus* (3.5 × 10^7^ cfu/kg)*, Bifidobacterium bifidum* (3.5 × 10^7^ cfu/kg) and *Saccharomyces cerevisiae* (2 × 10^9^ cfu/kg)*.*

### Experimental procedures and collections

Animals were weighed at the beginning and the end of each phase to determine average daily weight gain (DWG), average daily feed consumption (DFI), and to calculate the feed conversion (FCR). The left-overs were collected daily, weighed and subtracted from the quantity supplied to the animals.

During the first 14 days of the experiment, the occurrence of diarrhea was monitored daily by visual observation, always by the same observer. Fecal consistency was assessed according to the following scores: 1—normal feces; 2—pasty feces; and 3—liquid feces. Feces assigned with scores 1 and 2 were considered normal and feces with score 3 were considered diarrhea. The frequency of diarrhea was calculated based on the number of observation days. The frequency of fecal scores 1, 2 and 3 was the percentage of days that piglets presented these fecal scores in each pen. The calculation was performed as follows: Frequency of feces scores 1 or 2 or 3 (%) = [[(P1 × D) + (P2 × D) + (Pn × D)]/n/TD × 100], where P (1, 2 … N) = represents each piglet inside the pen (n); D = number of days that each piglet showed fecal scores 1, 2 or 3 within a pen; TD = total number of days on which the diarrhea scores were monitored^56^.

At 50 days of age, 2 mL of blood were collected to perform a blood count. The collection was performed through the jugular vein of one piglet per repetition—animal weighing closest to the average weight of the group in each pen. An automatic cell counter (Vet Scan HM 5; Abaxis) was used to evaluate hemoglobin, hematocrit, erythrocytes and leukocytes, and the ratio between neutrophils to lymphocytes was calculated.

At 50 days of age, a pool of fresh feces from 3 animals per repetition was swabbed to isolate *Escherichia coli*. These swabs were striated in Petri dishes containing MacConkey agar (Merck), incubated at 37 °C for 24 h to count the colony forming units. From this same pool, 2 g of feces were used for the sequencing of the microbiota by Illumina MiSeq. The samples were identified and frozen at − 20 °C for further analysis.

### DNA extraction, PCR amplification and sequencing

The feces pool samples were placed in a sterile 1.5 mL tube and sent to Neoprospecta Microbiome Technologies (Florianópolis-SC, Brazil). All procedures were performed according to the methodology previously described^57^. Sample preparation and sequencing were performed by Neoprospecta Microbiome Technologies. For total DNA extraction, the commercial QIAamp DNA Stool Mini Kit (QIAGEN, Hilden, Germany) was used according to the manufacturer's instructions. It consisted of the V3/V4 regions of the 16S rRNA gene, which were amplified using primers 341F (5′-CCTACGGGRSGCAGCAG-3′) and 806R (5′-GGACTACHVGGGTWTCTAAT-3′), with Illumina adapters, necessary for sequencing. The amplification was performed in 35 cycles at 50 °C of the annealing temperature, which was tripled for each sample. The sequencing was performed by Illumina MiSeq using V2 kits, with runs of 300 single-ended nucleotides.

### Sequence analysis

Read quality was assessed using the FastQC software (version 0.11.5) (https://www.bioinformatics.babraham.ac.uk/projects/fastqc/). Low quality reads and adapters were removed using the Trimmomatic program. The following steps were implemented using QIIME2 software (v. 2020.2)^58^ (https://qiime2.org/). The reads were subjected to a Denoising approach for low quality sequence removal, sequencing error correction, chimera removal and identification of amplicon sequence variants (ASVs) using the DADA2 method with default parameters, and 290 truncated read length. Taxonomy was attributed to ASVs using the SILVA database (v. 132), with 97% correspondence. Rare ASVs below a frequency of 0.1% in the samples were removed.

### Statistical analysis

The experimental design was completely randomized with three treatments (without additives, probiotics and a blend of functional oils), five repetitions per treatment (pens) and 15 piglets per repetition. The variables of performance, frequency of diarrhea, blood and *E. coli* quantification were subjected to an analysis of variance with 5% significance level, and means were tested by Tukey, using the SAS statistical program. Relative abundance, alpha rarefaction, alpha (Chao-1, Shannon and Simpson) and beta diversity indices were performed using the R program (v. 3.6.1) (https://www.R-project.org/) and plyr (v. 1.8.4)^59^, reshape2 (v. 1.4.3)^59^ and phyloseq packages (v. 1.14.0)^60^. Beta diversity was estimated after normalization by centered log-ratio using the DESeq2 R package (v. 1.26.0). After normalization, a principal coordinate analysis (PCoA) was performed using the Bray–Curtis dissimilarity index by vegan (v. 2.4.1)^61^ and heatmaps (v. 1.8.0) packages^61^. Alpha diversity and relative abundance were tested using the Kruskal–Wallis test. The Permutational Multivariate Analysis of Variance (Adonis at 999 permutations) was performed based on beta diversity, the assumption of homogeneity of variances was checked (*p* > 0.05) using the R vegan package. Differential abundances statistical was calculated using the DESeq2 R package^62^, aggregating the data at Family level.

## Supplementary Information

Supplementary Figure S1.

Supplementary Figure S2.

Supplementary Table S1.

Supplementary Table S2.

Supplementary Table S3.

## Data Availability

The raw sequences related to this article have been deposited at the Sequence Read Archive (SRA) under the BioProject ID PRJNA752610.
