# Supplementary figures and images for: Effect of functional oils or probiotics on performance and microbiota profile of newly weaned piglets
Source: Sci Rep. 2021 Sep 30;11:19457. doi: 10.1038/s41598-021-98549-w (PMC8484476; doi:10.1038/s41598-021-98549-w)

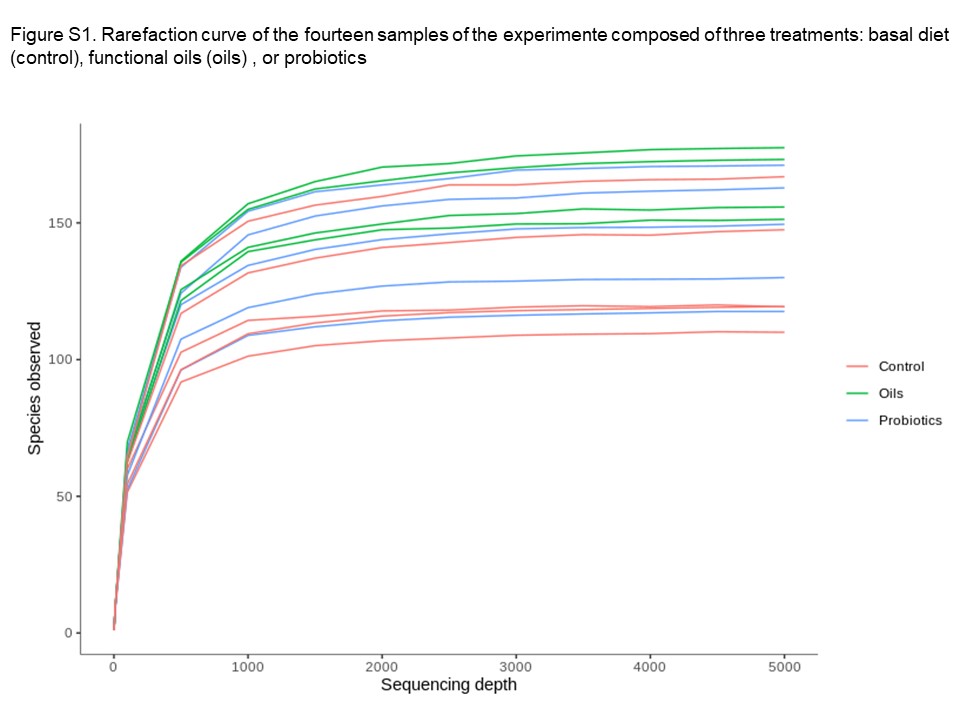

Supplement: Supplementary file 1 — Supplementary Figure S1. [file 41598_2021_98549_MOESM1_ESM.jpeg]

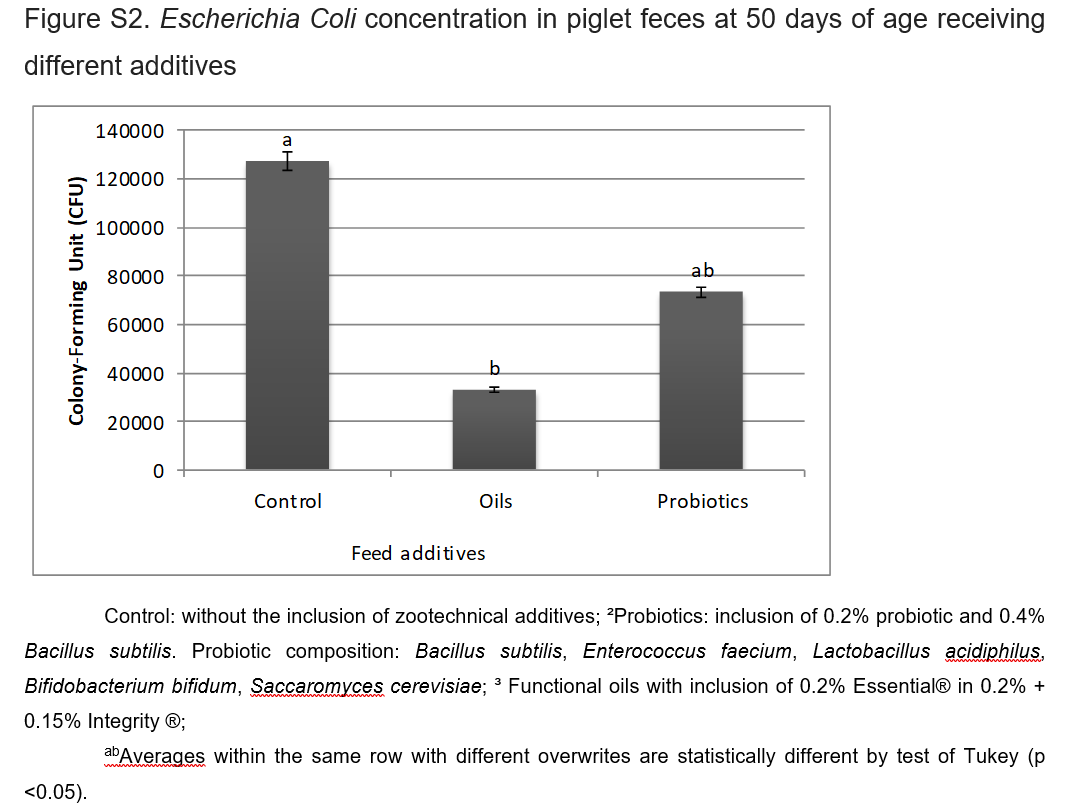

Supplement: Supplementary file 2 — Supplementary Figure S2. [file 41598_2021_98549_MOESM2_ESM.png]
