# Supplementary material for: Effect of functional oils or probiotics on performance and microbiota profile of newly weaned piglets
Source: Sci Rep. 2021 Sep 30;11:19457. doi: 10.1038/s41598-021-98549-w (PMC8484476; doi:10.1038/s41598-021-98549-w)
Supplement: Supplementary file 3 — Supplementary Table S1. [file 41598_2021_98549_MOESM3_ESM.doc]

**Supplementary Table S1.** Reads number that passed through each step of the quality control for the fifteen samples of the experiment composed of three feed additives, basal diet (control), funcional oils (oils), or Probiotics.

| **Sample id** | **Raw data** | **Filtered** | **Denoised** | **Non-chimeric** | **Non-rare** | **Treatment** |
| --- | --- | --- | --- | --- | --- | --- |
| 181113520743-1-1-1 | 114735 | 104376 | 85818 | 80076 | 79467 | Oils |
| 181113520744-1-1-1 | 120895 | 109479 | 95367 | 89960 | 89651 | Probiotics |
| 181113520746-1-1-1 | 100502 | 91263 | 78574 | 75338 | 74824 | Oils |
| 181113520747-1-1-1 | 96059 | 83542 | 73782 | 55246 | 54694 | Control |
| 181113520749-1-1-1 | 92071 | 83806 | 69410 | 62233 | 61532 | Probiotics |
| 181113520750-1-1-1 | 92948 | 82713 | 70171 | 59638 | 59485 | Control |
| 181113520751-1-1-1 | 118066 | 106326 | 92716 | 73696 | 73374 | Probiotics |
| 181113520752-1-1-1 | 106856 | 97730 | 84318 | 74525 | 74139 | Oils |
| 181113520754-1-1-1 | 105837 | 96475 | 85212 | 72571 | 71987 | Control |
| 181113520755-1-1-1 | 84557 | 77308 | 68883 | 61903 | 61491 | Probiotics |
| 181113520756-1-1-1 | 108242 | 96964 | 83083 | 78408 | 78005 | Oils |
| 181113520758-1-1-1 | 87856 | 79741 | 65579 | 54996 | 54434 | Probiotics |
| 181113520759-1-1-1 | 111268 | 101191 | 88365 | 78985 | 78460 | Control |
| **181113520761-1-1-1** | **22074** | **19481** | **13689** | **13159** | **12704** | **Oils** |
| 181113520762-1-1-1 | 65701 | 59793 | 48619 | 43679 | 43184 | Control |
