# Supplementary material for: Effect of functional oils or probiotics on performance and microbiota profile of newly weaned piglets
Source: Sci Rep. 2021 Sep 30;11:19457. doi: 10.1038/s41598-021-98549-w (PMC8484476; doi:10.1038/s41598-021-98549-w)
Supplement: Supplementary file 4 — Supplementary Table S2. [file 41598_2021_98549_MOESM4_ESM.docx]

**Table S2. Relative abundance of phylum, class, family and genus present in the intestinal microbiota of weaned piglets.**

| **Phylum** | Control (Mean) | Control (SD) | Oils (Mean) | Oils (SD) | Probiotic (Mean) | Probiotic (SD) | Kruskal-Wallis chi-squared | P-value |
| --- | --- | --- | --- | --- | --- | --- | --- | --- |
| Euryarchaeota | 0,0000 | 0,0000 | 0,0005 | 0,0010 | 0,0000 | 0,0008 | 3,0204 | 0,2209 |
| Actinobacteria | 0,0176 | 0,0060 | 0,0154 | 0,0132 | 0,0278 | 0,0181 | 1,1400 | 0,5655 |
| Bacteroidetes | 0,1977 | 0,0491 | 0,1799 | 0,0390 | 0,1623 | 0,0295 | 1,9629 | 0,3748 |
| Cyanobacteria | 0,0000 | 0,0000 | 0,0000 | 0,0009 | 0,0000 | 0,0014 | 2,2072 | 0,3317 |
| Epsilonbacteraeota | 0,0000 | 0,0012 | 0,0000 | 0,0005 | 0,0000 | 0,0015 | 0,4494 | 0,7988 |
| Fibrobacteres | 0,0000 | 0,0000 | 0,0000 | 0,0004 | 0,0000 | 0,0000 | 2,5000 | 0,2865 |
| Firmicutes | 0,7669 | 0,0546 | 0,7741 | 0,0527 | 0,8178 | 0,0330 | 1,9714 | 0,3732 |
| Patescibacteria | 0,0000 | 0,0007 | 0,0000 | 0,0000 | 0,0000 | 0,0000 | 1,8000 | 0,4066 |
| Proteobacteria | 0,0019 | 0,0012 | 0,0045 | 0,0056 | 0,0014 | 0,0092 | 0,5469 | 0,7608 |
| Spirochaetes | 0,0016 | 0,0010 | 0,0021 | 0,0020 | 0,0008 | 0,0019 | 3,4793 | 0,1756 |
| Tenericutes | 0,0142 | 0,0107 | 0,0189 | 0,0084 | 0,0052 | 0,0027 | 2,5657 | 0,2772 |
| **Class** |  |  |  |  |  |  |  |  |
| Methanobacteria | 0,0000 | 0,0000 | 0,0005 | 0,0010 | 0,0000 | 0,0008 | 3,0204 | 0,2209 |
| Actinobacteria | 0,0000 | 0,0005 | 0,0027 | 0,0033 | 0,0014 | 0,0089 | 3,1767 | 0,2043 |
| Coriobacteriia | 0,0165 | 0,0060 | 0,0114 | 0,0114 | 0,0204 | 0,0110 | 1,0714 | 0,5853 |
| Bacteroidia | 0,1977 | 0,0491 | 0,1799 | 0,0390 | 0,1623 | 0,0295 | 1,9629 | 0,3748 |
| Melainabacteria | 0,0000 | 0,0000 | 0,0000 | 0,0009 | 0,0000 | 0,0014 | 2,2072 | 0,3317 |
| Campylobacteria | 0,0000 | 0,0012 | 0,0000 | 0,0005 | 0,0000 | 0,0015 | 0,4494 | 0,7988 |
| Fibrobacteria | 0,0000 | 0,0000 | 0,0000 | 0,0004 | 0,0000 | 0,0000 | 2,5000 | 0,2865 |
| Bacilli | 0,0830 | 0,0728 | 0,0451 | 0,0263 | 0,0447 | 0,0174 | 3,2657 | 0,1954 |
| Clostridia | 0,5686 | 0,0528 | 0,6385 | 0,0787 | 0,6532 | 0,0448 | 3,1029 | 0,2119 |
| Erysipelotrichia | 0,0507 | 0,0265 | 0,0647 | 0,0254 | 0,0472 | 0,0148 | 0,6000 | 0,7408 |
| Negativicutes | 0,0545 | 0,0391 | 0,0330 | 0,0208 | 0,0623 | 0,0297 | 1,9857 | 0,3705 |
| Saccharimonadia | 0,0000 | 0,0007 | 0,0000 | 0,0000 | 0,0000 | 0,0000 | 1,8000 | 0,4066 |
| Deltaproteobacteria | 0,0000 | 0,0012 | 0,0005 | 0,0009 | 0,0000 | 0,0013 | 0,5110 | 0,7745 |
| Gammaproteobacteria | 0,0000 | 0,0010 | 0,0041 | 0,0047 | 0,0014 | 0,0080 | 1,4741 | 0,4785 |
| Spirochaetia | 0,0016 | 0,0010 | 0,0021 | 0,0020 | 0,0008 | 0,0019 | 3,4793 | 0,1756 |
| Mollicutes | 0,0142 | 0,0107 | 0,0189 | 0,0084 | 0,0052 | 0,0027 | 2,5657 | 0,2772 |
| Unknown | 0,0000 | 0,0000 | 0,0000 | 0,0000 | 0,0000 | 0,0011 | 1,8000 | 0,4066 |
| **Family** |  |  |  |  |  |  |  |  |
| Methanobacteriaceae | 0,0000 | 0,0000 | 0,0005 | 0,0010 | 0,0000 | 0,0008 | 3,0204 | 0,2209 |
| Bifidobacteriaceae | 0,0000 | 0,0005 | 0,0027 | 0,0033 | 0,0014 | 0,0089 | 3,1767 | 0,2043 |
| Atopobiaceae | 0,0081 | 0,0048 | 0,0053 | 0,0082 | 0,0093 | 0,0092 | 0,8657 | 0,6487 |
| Coriobacteriaceae | 0,0050 | 0,0019 | 0,0045 | 0,0028 | 0,0066 | 0,0014 | 3,3429 | 0,1880 |
| Eggerthellaceae | 0,0033 | 0,0018 | 0,0034 | 0,0012 | 0,0024 | 0,0017 | 1,2628 | 0,5319 |
| Bacteroidaceae | 0,0000 | 0,0011 | 0,0000 | 0,0000 | 0,0000 | 0,0000 | 1,8000 | 0,4066 |
| Bacteroidales RF16 group | 0,0000 | 0,0000 | 0,0000 | 0,0023 | 0,0000 | 0,0000 | 2,5000 | 0,2865 |
| Muribaculaceae | 0,0968 | 0,0216 | 0,0818 | 0,0330 | 0,0540 | 0,0180 | 4,4857 | 0,1062 |
| p-251-o5 | 0,0000 | 0,0022 | 0,0035 | 0,0083 | 0,0000 | 0,0000 | 6,0977 | 0,0474 |
| p-2534-18B5 gut group | 0,0000 | 0,0014 | 0,0000 | 0,0000 | 0,0000 | 0,0018 | 0,8769 | 0,6450 |
| Prevotellaceae | 0,0603 | 0,0370 | 0,0801 | 0,0167 | 0,0778 | 0,0241 | 0,3600 | 0,8353 |
| Rikenellaceae | 0,0140 | 0,0067 | 0,0178 | 0,0085 | 0,0130 | 0,0049 | 0,5000 | 0,7788 |
| Tannerellaceae | 0,0000 | 0,0073 | 0,0034 | 0,0023 | 0,0032 | 0,0021 | 1,7403 | 0,4189 |
| uncultured | 0,0000 | 0,0038 | 0,0000 | 0,0015 | 0,0017 | 0,0015 | 2,1804 | 0,3362 |
| Gastranaerophilales uncultured bacterium | 0,0000 | 0,0000 | 0,0000 | 0,0009 | 0,0000 | 0,0015 | 1,2019 | 0,5483 |
| Campylobacteraceae | 0,0000 | 0,0012 | 0,0000 | 0,0005 | 0,0000 | 0,0015 | 0,4494 | 0,7988 |
| Fibrobacteraceae | 0,0000 | 0,0000 | 0,0000 | 0,0004 | 0,0000 | 0,0000 | 2,5000 | 0,2865 |
| Enterococcaceae | 0,0000 | 0,0000 | 0,0000 | 0,0011 | 0,0000 | 0,0000 | 2,5000 | 0,2865 |
| Lactobacillaceae | 0,0796 | 0,0713 | 0,0414 | 0,0269 | 0,0439 | 0,0210 | 3,2657 | 0,1954 |
| Streptococcaceae | 0,0041 | 0,0030 | 0,0046 | 0,0027 | 0,0030 | 0,0051 | 0,1124 | 0,9453 |
| Christensenellaceae | 0,0111 | 0,0119 | 0,0096 | 0,0053 | 0,0013 | 0,0166 | 1,9889 | 0,3699 |
| Clostridiaceae 1 | 0,0263 | 0,0131 | 0,0405 | 0,0207 | 0,0355 | 0,0223 | 2,3829 | 0,3038 |
| Clostridiales vadinBB60 group | 0,0000 | 0,0000 | 0,0005 | 0,0007 | 0,0009 | 0,0019 | 3,8573 | 0,1453 |
| Defluviitaleaceae | 0,0000 | 0,0005 | 0,0000 | 0,0000 | 0,0000 | 0,0000 | 1,8000 | 0,4066 |
| Family XIII | 0,0265 | 0,0077 | 0,0386 | 0,0118 | 0,0165 | 0,0060 | 5,7114 | 0,0575 |
| Lachnospiraceae | 0,2063 | 0,0183 | 0,2028 | 0,0247 | 0,1863 | 0,0175 | 1,0029 | 0,6057 |
| Peptococcaceae | 0,0000 | 0,0020 | 0,0005 | 0,0010 | 0,0024 | 0,0015 | 2,7173 | 0,2570 |
| Peptostreptococcaceae | 0,0107 | 0,0050 | 0,0169 | 0,0088 | 0,0222 | 0,0112 | 0,3857 | 0,8246 |
| Ruminococcaceae | 0,2903 | 0,0439 | 0,3194 | 0,0291 | 0,3735 | 0,0215 | 5,3229 | 0,0698 |
| Erysipelotrichaceae | 0,0507 | 0,0265 | 0,0647 | 0,0254 | 0,0472 | 0,0148 | 0,6000 | 0,7408 |
| Acidaminococcaceae | 0,0062 | 0,0046 | 0,0095 | 0,0031 | 0,0122 | 0,0060 | 2,8314 | 0,2428 |
| Veillonellaceae | 0,0500 | 0,0357 | 0,0236 | 0,0185 | 0,0430 | 0,0271 | 2,1429 | 0,3425 |
| Saccharimonadaceae | 0,0000 | 0,0007 | 0,0000 | 0,0000 | 0,0000 | 0,0000 | 1,8000 | 0,4066 |
| uncultured rumen bacterium | 0,0000 | 0,0012 | 0,0000 | 0,0005 | 0,0000 | 0,0013 | 0,0111 | 0,9945 |
| Desulfovibrionaceae | 0,0000 | 0,0000 | 0,0000 | 0,0009 | 0,0000 | 0,0000 | 2,5000 | 0,2865 |
| Succinivibrionaceae | 0,0000 | 0,0000 | 0,0041 | 0,0042 | 0,0014 | 0,0071 | 5,1588 | 0,0758 |
| Enterobacteriaceae | 0,0000 | 0,0010 | 0,0000 | 0,0007 | 0,0000 | 0,0009 | 0,4673 | 0,7916 |
| Spirochaetaceae | 0,0016 | 0,0010 | 0,0021 | 0,0020 | 0,0008 | 0,0019 | 3,4793 | 0,1756 |
| metagenome | 0,0016 | 0,0030 | 0,0000 | 0,0013 | 0,0000 | 0,0014 | 1,3621 | 0,5061 |
| Mollicutes RF39 uncultured bacterium | 0,0012 | 0,0031 | 0,0010 | 0,0017 | 0,0000 | 0,0015 | 0,4105 | 0,8144 |
| uncultured Lachnospiraceae bacterium | 0,0000 | 0,0045 | 0,0010 | 0,0017 | 0,0000 | 0,0000 | 2,8869 | 0,2361 |
| uncultured organism | 0,0000 | 0,0007 | 0,0000 | 0,0006 | 0,0000 | 0,0000 | 1,2019 | 0,5483 |
| unidentified rumen bacterium RF39 | 0,0000 | 0,0000 | 0,0000 | 0,0016 | 0,0000 | 0,0000 | 2,5000 | 0,2865 |
| Unknown | 0,0075 | 0,0072 | 0,0181 | 0,0086 | 0,0052 | 0,0031 | 3,9344 | 0,1399 |
| **Genus** |  |  |  |  |  |  |  |  |
| Methanobrevibacter | 0,0000 | 0,0000 | 0,0005 | 0,0010 | 0,0000 | 0,0008 | 3,0204 | 0,2209 |
| Bifidobacterium | 0,0000 | 0,0005 | 0,0027 | 0,0033 | 0,0014 | 0,0089 | 3,1767 | 0,2043 |
| Libanicoccus | 0,0025 | 0,0028 | 0,0000 | 0,0016 | 0,0000 | 0,0035 | 1,0381 | 0,5951 |
| Atopobiaceae metagenome | 0,0000 | 0,0000 | 0,0000 | 0,0037 | 0,0000 | 0,0000 | 2,5000 | 0,2865 |
| Olsenella | 0,0027 | 0,0062 | 0,0000 | 0,0085 | 0,0082 | 0,0103 | 1,2636 | 0,5316 |
| Atopobiaceae uncultured | 0,0000 | 0,0010 | 0,0000 | 0,0008 | 0,0000 | 0,0006 | 0,1177 | 0,9429 |
| Collinsella | 0,0050 | 0,0019 | 0,0045 | 0,0028 | 0,0066 | 0,0014 | 3,3429 | 0,1880 |
| Enterorhabdus | 0,0017 | 0,0012 | 0,0021 | 0,0014 | 0,0015 | 0,0013 | 0,3747 | 0,8292 |
| Senegalimassilia | 0,0000 | 0,0011 | 0,0006 | 0,0008 | 0,0000 | 0,0009 | 0,3066 | 0,8579 |
| Slackia | 0,0000 | 0,0004 | 0,0000 | 0,0002 | 0,0000 | 0,0000 | 1,2019 | 0,5483 |
| Eggerthellaceae uncultured | 0,0000 | 0,0004 | 0,0008 | 0,0010 | 0,0000 | 0,0007 | 2,0172 | 0,3647 |
| Bacteroides | 0,0000 | 0,0011 | 0,0000 | 0,0000 | 0,0000 | 0,0000 | 1,8000 | 0,4066 |
| Bacteroidales RF16 group uncultured bacterium | 0,0000 | 0,0000 | 0,0000 | 0,0023 | 0,0000 | 0,0000 | 2,5000 | 0,2865 |
| Muribaculaceae metagenome | 0,0000 | 0,0017 | 0,0000 | 0,0000 | 0,0000 | 0,0018 | 1,9362 | 0,3798 |
| Muribaculaceae uncultured bacterium | 0,0171 | 0,0112 | 0,0164 | 0,0140 | 0,0049 | 0,0034 | 7,5629 | 0,0228 |
| uncultured Porphyromonadaceae bacterium | 0,0654 | 0,0250 | 0,0450 | 0,0238 | 0,0390 | 0,0099 | 0,9657 | 0,6170 |
| p-251-o5 uncultured bacterium | 0,0000 | 0,0022 | 0,0035 | 0,0083 | 0,0000 | 0,0000 | 6,0977 | 0,0474 |
| p-2534-18B5 gut group uncultured bacterium | 0,0000 | 0,0014 | 0,0000 | 0,0000 | 0,0000 | 0,0018 | 0,8769 | 0,6450 |
| Alloprevotella | 0,0025 | 0,0049 | 0,0071 | 0,0069 | 0,0053 | 0,0024 | 1,8068 | 0,4052 |
| Prevotella 1 | 0,0026 | 0,0018 | 0,0023 | 0,0013 | 0,0020 | 0,0050 | 0,1293 | 0,9374 |
| Prevotella 2 | 0,0066 | 0,0034 | 0,0068 | 0,0012 | 0,0050 | 0,0031 | 1,3057 | 0,5206 |
| Prevotella 7 | 0,0000 | 0,0054 | 0,0010 | 0,0015 | 0,0028 | 0,0050 | 0,6190 | 0,7338 |
| Prevotella 9 | 0,0073 | 0,0187 | 0,0141 | 0,0060 | 0,0257 | 0,0123 | 3,2400 | 0,1979 |
| Prevotellaceae NK3B31 group | 0,0116 | 0,0053 | 0,0165 | 0,0029 | 0,0102 | 0,0094 | 4,3829 | 0,1118 |
| Prevotellaceae UCG-001 | 0,0000 | 0,0000 | 0,0000 | 0,0000 | 0,0000 | 0,0013 | 1,8000 | 0,4066 |
| Prevotellaceae UCG-003 | 0,0110 | 0,0078 | 0,0118 | 0,0030 | 0,0097 | 0,0066 | 0,5914 | 0,7440 |
| Prevotellaceae uncultured | 0,0000 | 0,0006 | 0,0000 | 0,0007 | 0,0020 | 0,0034 | 8,5559 | 0,0139 |
| Alistipes | 0,0000 | 0,0000 | 0,0000 | 0,0005 | 0,0000 | 0,0000 | 2,5000 | 0,2865 |
| dgA-11 gut group | 0,0000 | 0,0015 | 0,0012 | 0,0017 | 0,0000 | 0,0000 | 2,6055 | 0,2718 |
| Rikenellaceae RC9 gut group | 0,0114 | 0,0068 | 0,0162 | 0,0067 | 0,0130 | 0,0049 | 0,7057 | 0,7027 |
| Parabacteroides | 0,0000 | 0,0073 | 0,0034 | 0,0023 | 0,0032 | 0,0021 | 1,7403 | 0,4189 |
| Gastranaerophilales uncultured bacterium | 0,0000 | 0,0000 | 0,0000 | 0,0009 | 0,0000 | 0,0015 | 1,2019 | 0,5483 |
| Campylobacter | 0,0000 | 0,0012 | 0,0000 | 0,0005 | 0,0000 | 0,0015 | 0,4494 | 0,7988 |
| Fibrobacter | 0,0000 | 0,0000 | 0,0000 | 0,0004 | 0,0000 | 0,0000 | 2,5000 | 0,2865 |
| Enterococcus | 0,0000 | 0,0000 | 0,0000 | 0,0011 | 0,0000 | 0,0000 | 2,5000 | 0,2865 |
| Lactobacillus | 0,0796 | 0,0713 | 0,0414 | 0,0269 | 0,0439 | 0,0210 | 3,2657 | 0,1954 |
| Streptococcus | 0,0041 | 0,0030 | 0,0046 | 0,0027 | 0,0030 | 0,0051 | 0,1124 | 0,9453 |
| Christensenellaceae R-7 group | 0,0111 | 0,0119 | 0,0096 | 0,0053 | 0,0013 | 0,0166 | 1,9889 | 0,3699 |
| Clostridium sensu stricto 1 | 0,0263 | 0,0135 | 0,0386 | 0,0198 | 0,0344 | 0,0207 | 1,9714 | 0,3732 |
| Clostridium sensu stricto 6 | 0,0000 | 0,0013 | 0,0012 | 0,0017 | 0,0010 | 0,0053 | 2,8737 | 0,2377 |
| Clostridiales vadinBB60 group uncultured organism | 0,0000 | 0,0000 | 0,0005 | 0,0007 | 0,0000 | 0,0013 | 2,6055 | 0,2718 |
| Defluviitaleaceae UCG-011 | 0,0000 | 0,0005 | 0,0000 | 0,0000 | 0,0000 | 0,0000 | 1,8000 | 0,4066 |
| [Eubacterium] nodatum group | 0,0000 | 0,0000 | 0,0007 | 0,0011 | 0,0000 | 0,0000 | 5,3846 | 0,0677 |
| Family XIII AD3011 group | 0,0128 | 0,0115 | 0,0180 | 0,0040 | 0,0121 | 0,0056 | 1,1829 | 0,5535 |
| Family XIII UCG-001 | 0,0014 | 0,0004 | 0,0013 | 0,0007 | 0,0000 | 0,0006 | 7,0044 | 0,0301 |
| Mogibacterium | 0,0108 | 0,0037 | 0,0164 | 0,0104 | 0,0061 | 0,0032 | 3,4714 | 0,1763 |
| [Eubacterium] eligens group | 0,0000 | 0,0027 | 0,0026 | 0,0031 | 0,0000 | 0,0018 | 0,7101 | 0,7011 |
| [Eubacterium] fissicatena group | 0,0000 | 0,0043 | 0,0000 | 0,0000 | 0,0000 | 0,0000 | 1,8000 | 0,4066 |
| [Eubacterium] hallii group | 0,0000 | 0,0012 | 0,0000 | 0,0000 | 0,0000 | 0,0000 | 1,8000 | 0,4066 |
| [Eubacterium] ruminantium group | 0,0000 | 0,0037 | 0,0032 | 0,0022 | 0,0000 | 0,0019 | 2,2313 | 0,3277 |
| [Ruminococcus] gauvreauii group | 0,0093 | 0,0094 | 0,0048 | 0,0085 | 0,0000 | 0,0036 | 2,4914 | 0,2877 |
| [Ruminococcus] torques group | 0,0066 | 0,0072 | 0,0082 | 0,0037 | 0,0102 | 0,0027 | 1,2885 | 0,5250 |
| Agathobacter | 0,0000 | 0,0069 | 0,0000 | 0,0000 | 0,0103 | 0,0147 | 4,0255 | 0,1336 |
| Blautia | 0,0459 | 0,0156 | 0,0295 | 0,0116 | 0,0374 | 0,0088 | 1,1829 | 0,5535 |
| Coprococcus 1 | 0,0298 | 0,0104 | 0,0127 | 0,0153 | 0,0157 | 0,0120 | 1,0000 | 0,6065 |
| Coprococcus 2 | 0,0000 | 0,0022 | 0,0000 | 0,0000 | 0,0000 | 0,0036 | 0,8769 | 0,6450 |
| Coprococcus 3 | 0,0091 | 0,0080 | 0,0129 | 0,0044 | 0,0085 | 0,0066 | 0,7257 | 0,6957 |
| Dorea | 0,0126 | 0,0132 | 0,0115 | 0,0038 | 0,0110 | 0,0053 | 0,7057 | 0,7027 |
| Fusicatenibacter | 0,0000 | 0,0068 | 0,0000 | 0,0021 | 0,0000 | 0,0033 | 0,8033 | 0,6692 |
| Howardella | 0,0000 | 0,0006 | 0,0000 | 0,0000 | 0,0000 | 0,0000 | 1,8000 | 0,4066 |
| Lachnoclostridium | 0,0116 | 0,0073 | 0,0000 | 0,0024 | 0,0000 | 0,0022 | 3,1937 | 0,2025 |
| Lachnospira | 0,0019 | 0,0066 | 0,0069 | 0,0040 | 0,0065 | 0,0045 | 1,4960 | 0,4733 |
| Lachnospiraceae AC2044 group | 0,0000 | 0,0000 | 0,0042 | 0,0041 | 0,0000 | 0,0038 | 5,4073 | 0,0670 |
| Lachnospiraceae FCS020 group | 0,0000 | 0,0041 | 0,0000 | 0,0008 | 0,0000 | 0,0026 | 0,4673 | 0,7916 |
| Lachnospiraceae ND3007 group | 0,0000 | 0,0039 | 0,0035 | 0,0055 | 0,0000 | 0,0035 | 1,0478 | 0,5922 |
| Lachnospiraceae NK3A20 group | 0,0000 | 0,0032 | 0,0000 | 0,0027 | 0,0000 | 0,0034 | 0,0111 | 0,9945 |
| Lachnospiraceae NK4A136 group | 0,0000 | 0,0048 | 0,0089 | 0,0052 | 0,0100 | 0,0047 | 2,4924 | 0,2876 |
| Lachnospiraceae UCG-004 | 0,0000 | 0,0016 | 0,0000 | 0,0000 | 0,0000 | 0,0000 | 1,8000 | 0,4066 |
| Lachnospiraceae XPB1014 group | 0,0000 | 0,0000 | 0,0000 | 0,0000 | 0,0000 | 0,0036 | 1,8000 | 0,4066 |
| Marvinbryantia | 0,0045 | 0,0026 | 0,0048 | 0,0013 | 0,0062 | 0,0034 | 3,6031 | 0,1650 |
| Moryella | 0,0000 | 0,0016 | 0,0000 | 0,0000 | 0,0000 | 0,0000 | 1,8000 | 0,4066 |
| Oribacterium | 0,0000 | 0,0028 | 0,0015 | 0,0032 | 0,0000 | 0,0027 | 0,6287 | 0,7303 |
| Roseburia | 0,0000 | 0,0027 | 0,0000 | 0,0000 | 0,0000 | 0,0089 | 2,2902 | 0,3182 |
| Syntrophococcus | 0,0000 | 0,0000 | 0,0000 | 0,0051 | 0,0000 | 0,0074 | 2,2072 | 0,3317 |
| Lachnospiraceae uncultured | 0,0020 | 0,0055 | 0,0047 | 0,0029 | 0,0017 | 0,0011 | 2,6490 | 0,2659 |
| Peptococcus | 0,0000 | 0,0020 | 0,0005 | 0,0010 | 0,0024 | 0,0015 | 2,7173 | 0,2570 |
| [Eubacterium] coprostanoligenes group | 0,0207 | 0,0128 | 0,0239 | 0,0107 | 0,0297 | 0,0096 | 0,1314 | 0,9364 |
| Butyricicoccus | 0,0000 | 0,0000 | 0,0009 | 0,0029 | 0,0034 | 0,0020 | 5,3296 | 0,0696 |
| Candidatus Soleaferrea | 0,0000 | 0,0011 | 0,0024 | 0,0019 | 0,0023 | 0,0017 | 3,5170 | 0,1723 |
| Caproiciproducens | 0,0000 | 0,0007 | 0,0000 | 0,0000 | 0,0000 | 0,0000 | 1,8000 | 0,4066 |
| Faecalibacterium | 0,0211 | 0,0111 | 0,0298 | 0,0162 | 0,0701 | 0,0259 | 6,9417 | 0,0311 |
| Fournierella | 0,0107 | 0,0073 | 0,0127 | 0,0069 | 0,0117 | 0,0080 | 0,3714 | 0,8305 |
| Intestinimonas | 0,0033 | 0,0023 | 0,0023 | 0,0029 | 0,0000 | 0,0037 | 0,0586 | 0,9711 |
| Negativibacillus | 0,0034 | 0,0016 | 0,0032 | 0,0022 | 0,0035 | 0,0009 | 0,2029 | 0,9035 |
| Oscillibacter | 0,0000 | 0,0000 | 0,0000 | 0,0007 | 0,0000 | 0,0041 | 2,4894 | 0,2880 |
| Oscillospira | 0,0074 | 0,0090 | 0,0121 | 0,0045 | 0,0040 | 0,0073 | 1,3146 | 0,5182 |
| Pygmaiobacter | 0,0000 | 0,0000 | 0,0000 | 0,0000 | 0,0000 | 0,0005 | 1,8000 | 0,4066 |
| Ruminiclostridium 5 | 0,0028 | 0,0033 | 0,0070 | 0,0036 | 0,0053 | 0,0017 | 1,8029 | 0,4060 |
| Ruminiclostridium 6 | 0,0016 | 0,0019 | 0,0018 | 0,0009 | 0,0000 | 0,0005 | 5,0979 | 0,0782 |
| Ruminiclostridium 9 | 0,0000 | 0,0048 | 0,0043 | 0,0035 | 0,0033 | 0,0028 | 0,4403 | 0,8024 |
| Ruminococcaceae NK4A214 group | 0,0234 | 0,0124 | 0,0285 | 0,0062 | 0,0337 | 0,0090 | 2,7857 | 0,2484 |
| Ruminococcaceae UCG-002 | 0,0117 | 0,0094 | 0,0200 | 0,0053 | 0,0168 | 0,0127 | 0,6000 | 0,7408 |
| Ruminococcaceae UCG-003 | 0,0000 | 0,0000 | 0,0000 | 0,0010 | 0,0000 | 0,0000 | 2,5000 | 0,2865 |
| Ruminococcaceae UCG-004 | 0,0000 | 0,0020 | 0,0022 | 0,0026 | 0,0000 | 0,0029 | 0,8203 | 0,6635 |
| Ruminococcaceae UCG-005 | 0,0402 | 0,0142 | 0,0555 | 0,0236 | 0,0417 | 0,0054 | 1,2857 | 0,5258 |
| Ruminococcaceae UCG-008 | 0,0084 | 0,0069 | 0,0066 | 0,0046 | 0,0035 | 0,0042 | 1,3115 | 0,5190 |
| Ruminococcaceae UCG-009 | 0,0018 | 0,0028 | 0,0021 | 0,0006 | 0,0018 | 0,0005 | 1,0714 | 0,5853 |
| Ruminococcaceae UCG-010 | 0,0030 | 0,0031 | 0,0023 | 0,0010 | 0,0029 | 0,0019 | 0,1117 | 0,9457 |
| Ruminococcaceae UCG-013 | 0,0000 | 0,0012 | 0,0000 | 0,0006 | 0,0000 | 0,0004 | 1,0591 | 0,5889 |
| Ruminococcaceae UCG-014 | 0,0264 | 0,0153 | 0,0338 | 0,0093 | 0,0369 | 0,0128 | 0,1800 | 0,9139 |
| Ruminococcus 1 | 0,0048 | 0,0028 | 0,0074 | 0,0052 | 0,0129 | 0,0081 | 5,5714 | 0,0617 |
| Ruminococcus 2 | 0,0060 | 0,0039 | 0,0067 | 0,0078 | 0,0138 | 0,0068 | 5,4527 | 0,0655 |
| Subdoligranulum | 0,0388 | 0,0427 | 0,0435 | 0,0075 | 0,0603 | 0,0111 | 3,7429 | 0,1539 |
| Ruminococcaceae uncultured | 0,0022 | 0,0058 | 0,0019 | 0,0018 | 0,0016 | 0,0031 | 1,0118 | 0,6030 |
| [Anaerorhabdus] furcosa group | 0,0000 | 0,0010 | 0,0012 | 0,0008 | 0,0006 | 0,0005 | 2,0820 | 0,3531 |
| Catenibacterium | 0,0051 | 0,0010 | 0,0054 | 0,0082 | 0,0180 | 0,0076 | 6,3514 | 0,0418 |
| Catenisphaera | 0,0070 | 0,0064 | 0,0068 | 0,0044 | 0,0033 | 0,0028 | 1,3000 | 0,5220 |
| Erysipelotrichaceae UCG-003 | 0,0000 | 0,0007 | 0,0000 | 0,0006 | 0,0000 | 0,0010 | 0,0111 | 0,9945 |
| Erysipelotrichaceae UCG-004 | 0,0000 | 0,0012 | 0,0000 | 0,0010 | 0,0000 | 0,0006 | 0,0830 | 0,9594 |
| Erysipelotrichaceae UCG-006 | 0,0000 | 0,0013 | 0,0010 | 0,0015 | 0,0014 | 0,0014 | 0,2541 | 0,8807 |
| Holdemanella | 0,0172 | 0,0127 | 0,0155 | 0,0075 | 0,0114 | 0,0070 | 1,0029 | 0,6057 |
| Sharpea | 0,0000 | 0,0007 | 0,0000 | 0,0007 | 0,0000 | 0,0027 | 0,0111 | 0,9945 |
| Solobacterium | 0,0129 | 0,0082 | 0,0056 | 0,0046 | 0,0072 | 0,0063 | 2,6914 | 0,2604 |
| Erysipelotrichaceae uncultured | 0,0141 | 0,0065 | 0,0142 | 0,0152 | 0,0047 | 0,0023 | 7,8600 | 0,0196 |
| Acidaminococcus | 0,0000 | 0,0014 | 0,0000 | 0,0007 | 0,0000 | 0,0029 | 0,7004 | 0,7045 |
| Phascolarctobacterium | 0,0046 | 0,0046 | 0,0088 | 0,0034 | 0,0106 | 0,0041 | 2,9314 | 0,2309 |
| Allisonella | 0,0000 | 0,0015 | 0,0000 | 0,0000 | 0,0000 | 0,0008 | 1,6706 | 0,4337 |
| Anaerovibrio | 0,0004 | 0,0020 | 0,0026 | 0,0025 | 0,0017 | 0,0047 | 0,6276 | 0,7307 |
| Dialister | 0,0000 | 0,0017 | 0,0012 | 0,0021 | 0,0027 | 0,0062 | 2,0253 | 0,3632 |
| Megasphaera | 0,0389 | 0,0291 | 0,0121 | 0,0150 | 0,0281 | 0,0237 | 1,9822 | 0,3712 |
| Mitsuokella | 0,0013 | 0,0018 | 0,0014 | 0,0043 | 0,0000 | 0,0047 | 0,0977 | 0,9523 |
| Schwartzia | 0,0000 | 0,0011 | 0,0000 | 0,0000 | 0,0000 | 0,0008 | 0,8769 | 0,6450 |
| Selenomonas | 0,0000 | 0,0000 | 0,0000 | 0,0000 | 0,0000 | 0,0021 | 1,8000 | 0,4066 |
| Veillonellaceae uncultured | 0,0000 | 0,0130 | 0,0000 | 0,0000 | 0,0000 | 0,0000 | 1,8000 | 0,4066 |
| Candidatus Saccharimonas | 0,0000 | 0,0007 | 0,0000 | 0,0000 | 0,0000 | 0,0000 | 1,8000 | 0,4066 |
| uncultured rumen bacterium | 0,0000 | 0,0012 | 0,0000 | 0,0005 | 0,0000 | 0,0013 | 0,0111 | 0,9945 |
| Desulfovibrio | 0,0000 | 0,0000 | 0,0000 | 0,0009 | 0,0000 | 0,0000 | 2,5000 | 0,2865 |
| Succinivibrio | 0,0000 | 0,0000 | 0,0041 | 0,0042 | 0,0014 | 0,0071 | 5,1588 | 0,0758 |
| Escherichia-Shigella | 0,0000 | 0,0010 | 0,0000 | 0,0007 | 0,0000 | 0,0009 | 0,4673 | 0,7916 |
| Treponema 2 | 0,0016 | 0,0010 | 0,0021 | 0,0020 | 0,0008 | 0,0019 | 3,4793 | 0,1756 |
| Mollicutes RF39 metagenome | 0,0016 | 0,0030 | 0,0000 | 0,0013 | 0,0000 | 0,0014 | 1,3621 | 0,5061 |
| Mollicutes RF39 uncultured bacterium | 0,0012 | 0,0031 | 0,0010 | 0,0017 | 0,0000 | 0,0015 | 0,4105 | 0,8144 |
| uncultured Lachnospiraceae bacterium | 0,0000 | 0,0045 | 0,0010 | 0,0017 | 0,0000 | 0,0000 | 2,8869 | 0,2361 |
| Mollicutes RF39 uncultured organism | 0,0000 | 0,0007 | 0,0000 | 0,0006 | 0,0000 | 0,0000 | 1,2019 | 0,5483 |
| unidentified rumen bacterium RF39 | 0,0000 | 0,0000 | 0,0000 | 0,0016 | 0,0000 | 0,0000 | 2,5000 | 0,2865 |
| Unknown | 0,0946 | 0,0427 | 0,1430 | 0,0397 | 0,0914 | 0,0241 | 3,9514 | 0,1387 |

| **Phylum** | **Control (Average)** | **Control (SD)** | **Oils (Average)** | **Oils (SD)** | **Probiotics (Average)** | **Probiotics (SD)** | **Kruskal-Wallis chi-squared** | **P-value** |
| --- | --- | --- | --- | --- | --- | --- | --- | --- |
| Euryarchaeota | 0.0000 | 0.0000 | 0.0008 | 0.0010 | 0.0004 | 0.0008 | 3.0204 | 0.2209 |
| Actinobacteria | 0.0165 | 0.0060 | 0.0194 | 0.0132 | 0.0285 | 0.0181 | 1.1400 | 0.5655 |
| Bacteroidetes | 0.1759 | 0.0491 | 0.1482 | 0.0390 | 0.1552 | 0.0295 | 1.9629 | 0.3748 |
| Cyanobacteria | 0.0000 | 0.0000 | 0.0004 | 0.0009 | 0.0009 | 0.0014 | 2.2072 | 0.3317 |
| Epsilonbacteraeota | 0.0005 | 0.0012 | 0.0003 | 0.0005 | 0.0008 | 0.0015 | 0.4494 | 0.7988 |
| Firmicutes | 0.7912 | 0.0546 | 0.8165 | 0.0527 | 0.8018 | 0.0330 | 1.9714 | 0.3732 |
| Proteobacteria | 0.0013 | 0.0012 | 0.0053 | 0.0056 | 0.0051 | 0.0092 | 0.5469 | 0.7608 |
| Spirochaetes | 0.0010 | 0.0010 | 0.0028 | 0.0020 | 0.0013 | 0.0019 | 3.4793 | 0.1756 |
| Tenericutes | 0.0132 | 0.0107 | 0.0162 | 0.0084 | 0.0060 | 0.0027 | 2.1000 | 0.3499 |
| **Class** |  |  |  |  |  |  |  |  |
| Methanobacteria | 0.0000 | 0.0000 | 0.0008 | 0.0010 | 0.0004 | 0.0008 | 3.0204 | 0.2209 |
| Actinobacteria | 0.0002 | 0.0005 | 0.0031 | 0.0033 | 0.0070 | 0.0089 | 3.1767 | 0.2043 |
| Coriobacteriia | 0.0163 | 0.0060 | 0.0163 | 0.0114 | 0.0215 | 0.0110 | 1.0714 | 0.5853 |
| Bacteroidia | 0.1759 | 0.0491 | 0.1882 | 0.0390 | 0.1552 | 0.0295 | 1.9629 | 0.3748 |
| Melainabacteria | 0.0000 | 0.0000 | 0.0004 | 0.0009 | 0.0009 | 0.0014 | 2.2072 | 0.3317 |
| Campylobacteria | 0.0005 | 0.0012 | 0.0003 | 0.0005 | 0.0008 | 0.0015 | 0.4494 | 0.7988 |
| Bacilli | 0.0989 | 0.0728 | 0.0437 | 0.0263 | 0.0419 | 0.0174 | 3.2657 | 0.1954 |
| Clostridia | 0.5836 | 0.0528 | 0.6311 | 0.0787 | 0.6504 | 0.0448 | 3.1029 | 0.2119 |
| Erysipelotrichia | 0.0604 | 0.0265 | 0.0594 | 0.0254 | 0.0504 | 0.0148 | 0.6000 | 0.7408 |
| Negativicutes | 0.0482 | 0.0391 | 0.0322 | 0.0208 | 0.0586 | 0.0297 | 1.9857 | 0.3705 |
| Deltaproteobacteria | 0.0005 | 0.0012 | 0.0007 | 0.0009 | 0.0006 | 0.0013 | 0.5110 | 0.7745 |
| Gammaproteobacteria | 0.0007 | 0.0010 | 0.0046 | 0.0047 | 0.0045 | 0.0080 | 1.4741 | 0.4785 |
| Spirochaetia | 0.0010 | 0.0010 | 0.0028 | 0.0020 | 0.0013 | 0.0019 | 3.4793 | 0.1756 |
| Mollicutes | 0.0132 | 0.0107 | 0.0162 | 0.0084 | 0.0060 | 0.0027 | 2.1000 | 0.3499 |
| **Family** |  |  |  |  |  |  |  |  |
| Methanobacteriaceae | 0.0000 | 0.0000 | 0.0008 | 0.0010 | 0.0004 | 0.0008 | 3.0204 | 0.2209 |
| Bifidobacteriaceae | 0.0002 | 0.0005 | 0.0031 | 0.0033 | 0.0070 | 0.0089 | 3.1767 | 0.2043 |
| Atopobiaceae | 0.0085 | 0.0048 | 0.0073 | 0.0082 | 0.0124 | 0.0092 | 0.8657 | 0.6487 |
| Coriobacteriaceae | 0.0051 | 0.0019 | 0.0055 | 0.0028 | 0.0069 | 0.0014 | 3.3429 | 0.1880 |
| Eggerthellaceae | 0.0027 | 0.0018 | 0.0035 | 0.0012 | 0.0023 | 0.0017 | 1.2628 | 0.5319 |
| Muribaculaceae | 0.0935 | 0.0216 | 0.0813 | 0.0330 | 0.0565 | 0.0180 | 5.1257 | 0.0771 |
| p-251-o5 | 0.0010 | 0.0022 | 0.0063 | 0.0083 | 0.0000 | 0.0000 | 6.0977 | 0.0474 |
| p-2534-18B5 gut group | 0.0006 | 0.0014 | 0.0000 | 0.0000 | 0.0008 | 0.0018 | 0.8769 | 0.6450 |
| Prevotellaceae | 0.0606 | 0.0370 | 0.0734 | 0.0167 | 0.0788 | 0.0241 | 0.3600 | 0.8353 |
| Rikenellaceae | 0.0138 | 0.0067 | 0.0188 | 0.0085 | 0.0136 | 0.0049 | 0.5000 | 0.7788 |
| Tannerellaceae | 0.0032 | 0.0073 | 0.0031 | 0.0023 | 0.0029 | 0.0021 | 1.7403 | 0.4189 |
| uncultured | 0.0017 | 0.0038 | 0.0008 | 0.0015 | 0.0017 | 0.0015 | 2.1804 | 0.3362 |
| Gastranaerophilales uncultured bacterium | 0.0000 | 0.0000 | 0.0004 | 0.0009 | 0.0007 | 0.0015 | 1.2019 | 0.5483 |
| Campylobacteraceae | 0.0005 | 0.0012 | 0.0003 | 0.0005 | 0.0008 | 0.0015 | 0.4494 | 0.7988 |
| Lactobacillaceae | 0.0948 | 0.0713 | 0.0394 | 0.0269 | 0.0375 | 0.0210 | 3.2657 | 0.1954 |
| Streptococcaceae | 0.0041 | 0.0030 | 0.0038 | 0.0027 | 0.0044 | 0.0051 | 0.1124 | 0.9453 |
| Christensenellaceae | 0.0134 | 0.0119 | 0.0100 | 0.0053 | 0.0081 | 0.0166 | 1.9889 | 0.3699 |
| Clostridiaceae 1 | 0.0203 | 0.0131 | 0.0413 | 0.0207 | 0.0430 | 0.0223 | 2.3829 | 0.3038 |
| Clostridiales vadinBB60 group | 0.0000 | 0.0000 | 0.0006 | 0.0007 | 0.0014 | 0.0019 | 3.8573 | 0.1453 |
| Family XIII | 0.0295 | 0.0077 | 0.0343 | 0.0118 | 0.0196 | 0.0060 | 5.7114 | 0.0575 |
| Lachnospiraceae | 0.2028 | 0.0183 | 0.2035 | 0.0247 | 0.1918 | 0.0175 | 1.3000 | 0.5220 |
| Peptococcaceae | 0.0009 | 0.0020 | 0.0008 | 0.0010 | 0.0021 | 0.0015 | 2.7173 | 0.2570 |
| Peptostreptococcaceae | 0.0130 | 0.0050 | 0.0165 | 0.0088 | 0.0174 | 0.0112 | 0.3857 | 0.8246 |
| Ruminococcaceae | 0.3036 | 0.0439 | 0.3231 | 0.0291 | 0.3672 | 0.0215 | 5.3229 | 0.0698 |
| Erysipelotrichaceae | 0.0604 | 0.0265 | 0.0594 | 0.0254 | 0.0504 | 0.0148 | 0.6000 | 0.7408 |
| Acidaminococcaceae | 0.0062 | 0.0046 | 0.0105 | 0.0031 | 0.0111 | 0.0060 | 2.8314 | 0.2428 |
| Veillonellaceae | 0.0420 | 0.0357 | 0.0217 | 0.0185 | 0.0475 | 0.0271 | 2.1429 | 0.3425 |
| uncultured rumen bacterium | 0.0005 | 0.0012 | 0.0002 | 0.0005 | 0.0006 | 0.0013 | 0.0111 | 0.9945 |
| Succinivibrionaceae | 0.0000 | 0.0000 | 0.0043 | 0.0042 | 0.0041 | 0.0071 | 5.1588 | 0.0758 |
| Enterobacteriaceae | 0.0007 | 0.0010 | 0.0003 | 0.0007 | 0.0004 | 0.0009 | 0.4673 | 0.7916 |
| Spirochaetaceae | 0.0010 | 0.0010 | 0.0028 | 0.0020 | 0.0013 | 0.0019 | 3.4793 | 0.1756 |
| metagenome | 0.0021 | 0.0030 | 0.0006 | 0.0013 | 0.0006 | 0.0014 | 1.3621 | 0.5061 |
| Mollicutes RF39 uncultured bacterium | 0.0021 | 0.0031 | 0.0014 | 0.0017 | 0.0010 | 0.0015 | 0.4105 | 0.8144 |
| uncultured Lachnospiraceae bacterium | 0.0025 | 0.0045 | 0.0014 | 0.0017 | 0.0000 | 0.0000 | 2.8869 | 0.2361 |
| uncultured organism | 0.0003 | 0.0007 | 0.0003 | 0.0006 | 0.0000 | 0.0000 | 1.2019 | 0.5483 |
| Unknown | 0.0073 | 0.0072 | 0.0161 | 0.0086 | 0.0060 | 0.0031 | 3.3874 | 0.1838 |
| **Genus** |  |  |  |  |  |  |  |  |
| Methanobrevibacter | 0.0000 | 0.0000 | 0.0008 | 0.0010 | 0.0004 | 0.0008 | 3.0204 | 0.2209 |
| Bifidobacterium | 0.0002 | 0.0005 | 0.0031 | 0.0033 | 0.0070 | 0.0089 | 3.1767 | 0.2043 |
| Libanicoccus | 0.0027 | 0.0028 | 0.0008 | 0.0016 | 0.0025 | 0.0035 | 1.0381 | 0.5951 |
| Olsenella | 0.0053 | 0.0062 | 0.0043 | 0.0085 | 0.0094 | 0.0103 | 1.2636 | 0.5316 |
| Atopobiaceae uncultured | 0.0005 | 0.0010 | 0.0004 | 0.0008 | 0.0004 | 0.0006 | 0.1177 | 0.9429 |
| Collinsella | 0.0051 | 0.0019 | 0.0055 | 0.0028 | 0.0069 | 0.0014 | 3.3429 | 0.1880 |
| Enterorhabdus | 0.0018 | 0.0012 | 0.0019 | 0.0014 | 0.0013 | 0.0013 | 0.3747 | 0.8292 |
| Senegalimassilia | 0.0005 | 0.0011 | 0.0007 | 0.0008 | 0.0007 | 0.0009 | 0.3066 | 0.8579 |
| Slackia | 0.0002 | 0.0004 | 0.0001 | 0.0002 | 0.0000 | 0.0000 | 1.2019 | 0.5483 |
| Eggerthellaceae uncultured | 0.0002 | 0.0004 | 0.0009 | 0.0010 | 0.0003 | 0.0007 | 2.0172 | 0.3647 |
| Muribaculaceae metagenome | 0.0007 | 0.0017 | 0.0000 | 0.0000 | 0.0011 | 0.0018 | 1.9362 | 0.3798 |
| Muribaculaceae uncultured bacterium | 0.0178 | 0.0112 | 0.0221 | 0.0140 | 0.0050 | 0.0034 | 7.5629 | 0.0228 |
| uncultured Porphyromonadaceae bacterium | 0.0613 | 0.0250 | 0.0509 | 0.0238 | 0.0435 | 0.0099 | 0.9657 | 0.6170 |
| p-251-o5 uncultured bacterium | 0.0010 | 0.0022 | 0.0063 | 0.0083 | 0.0000 | 0.0000 | 6.0977 | 0.0474 |
| p-2534-18B5 gut group uncultured bacterium | 0.0006 | 0.0014 | 0.0000 | 0.0000 | 0.0008 | 0.0018 | 0.8769 | 0.6450 |
| Alloprevotella | 0.0038 | 0.0049 | 0.0089 | 0.0069 | 0.0053 | 0.0024 | 1.8068 | 0.4052 |
| Prevotella 1 | 0.0019 | 0.0018 | 0.0020 | 0.0013 | 0.0039 | 0.0050 | 0.0202 | 0.9900 |
| Prevotella 2 | 0.0048 | 0.0034 | 0.0069 | 0.0012 | 0.0047 | 0.0031 | 1.3057 | 0.5206 |
| Prevotella 7 | 0.0027 | 0.0054 | 0.0012 | 0.0015 | 0.0037 | 0.0050 | 0.6190 | 0.7338 |
| Prevotella 9 | 0.0156 | 0.0187 | 0.0137 | 0.0060 | 0.0269 | 0.0123 | 3.2400 | 0.1979 |
| Prevotellaceae NK3B31 group | 0.0093 | 0.0053 | 0.0165 | 0.0029 | 0.0117 | 0.0094 | 4.3829 | 0.1118 |
| Prevotellaceae UCG 003 | 0.0101 | 0.0078 | 0.0120 | 0.0030 | 0.0092 | 0.0066 | 0.5914 | 0.7440 |
| Prevotellaceae uncultured | 0.0003 | 0.0006 | 0.0004 | 0.0007 | 0.0036 | 0.0034 | 8.5559 | 0.0139 |
| DgA-11 gut group | 0.0007 | 0.0015 | 0.0014 | 0.0017 | 0.0000 | 0.0000 | 2.6055 | 0.2718 |
| Rikenellaceae RC9 gut group | 0.0131 | 0.0068 | 0.0171 | 0.0067 | 0.0136 | 0.0049 | 0.7057 | 0.7027 |
| Parabacteroides | 0.0032 | 0.0073 | 0.0031 | 0.0023 | 0.0029 | 0.0021 | 1.7403 | 0.4189 |
| Gastranaerophilales uncultured bacterium | 0.0000 | 0.0000 | 0.0004 | 0.0009 | 0.0007 | 0.0015 | 1.2019 | 0.5483 |
| Campylobacter | 0.0005 | 0.0012 | 0.0003 | 0.0005 | 0.0008 | 0.0015 | 0.4494 | 0.7988 |
| Lactobacillus | 0.0948 | 0.0713 | 0.0394 | 0.0269 | 0.0375 | 0.0210 | 3.2657 | 0.1954 |
| Streptococcus | 0.0041 | 0.0030 | 0.0038 | 0.0027 | 0.0044 | 0.0051 | 0.1124 | 0.9453 |
| Christensenellaceae R 7 group | 0.0134 | 0.0119 | 0.0100 | 0.0053 | 0.0081 | 0.0166 | 1.9889 | 0.3699 |
| Clostridium sensu stricto 1 | 0.0197 | 0.0135 | 0.0395 | 0.0198 | 0.0387 | 0.0207 | 1.9714 | 0.3732 |
| Clostridium sensu stricto 6 | 0.0006 | 0.0013 | 0.0014 | 0.0017 | 0.0043 | 0.0053 | 2.8737 | 0.2377 |
| Clostridiales vadinBB60 group uncultured | 0.0000 | 0.0000 | 0.0006 | 0.0007 | 0.0006 | 0.0013 | 2.6055 | 0.2718 |
| Family XIII AD3011 group | 0.0177 | 0.0115 | 0.0168 | 0.0040 | 0.0138 | 0.0056 | 1.1829 | 0.5535 |
| Family XIII UCG-001 | 0.0015 | 0.0004 | 0.0015 | 0.0007 | 0.0004 | 0.0006 | 7.0044 | 0.0301 |
| Mogibacterium | 0.0097 | 0.0037 | 0.0140 | 0.0104 | 0.0053 | 0.0032 | 3.4714 | 0.1763 |
| [Eubacterium] eligens group | 0.0012 | 0.0027 | 0.0027 | 0.0031 | 0.0012 | 0.0018 | 0.7101 | 0.7011 |
| [Eubacterium] ruminantium group | 0.0016 | 0.0037 | 0.0029 | 0.0022 | 0.0009 | 0.0019 | 2.2313 | 0.3277 |
| [Ruminococcus] gauvreauii group | 0.0091 | 0.0094 | 0.0068 | 0.0085 | 0.0016 | 0.0036 | 2.4914 | 0.2877 |
| [Ruminococcus] torques group | 0.0070 | 0.0072 | 0.0087 | 0.0037 | 0.0106 | 0.0027 | 1.2885 | 0.5250 |
| Agathobacter | 0.0031 | 0.0069 | 0.0000 | 0.0000 | 0.0124 | 0.0147 | 4.0255 | 0.1336 |
| Blautia | 0.0365 | 0.0156 | 0.0284 | 0.0116 | 0.0341 | 0.0088 | 1.1829 | 0.5535 |
| Coprococcus 1 | 0.0270 | 0.0104 | 0.0155 | 0.0153 | 0.0194 | 0.0120 | 1.0000 | 0.6065 |
| Coprococcus 2 | 0.0010 | 0.0022 | 0.0000 | 0.0000 | 0.0016 | 0.0036 | 0.8769 | 0.6450 |
| Coprococcus 3 | 0.0107 | 0.0080 | 0.0132 | 0.0044 | 0.0114 | 0.0066 | 0.7257 | 0.6957 |
| Dorea | 0.0169 | 0.0132 | 0.0101 | 0.0038 | 0.0121 | 0.0053 | 0.7057 | 0.7027 |
| Fusicatenibacter | 0.0048 | 0.0068 | 0.0011 | 0.0021 | 0.0024 | 0.0033 | 0.8033 | 0.6692 |
| Lachnoclostridium | 0.0079 | 0.0073 | 0.0012 | 0.0024 | 0.0010 | 0.0022 | 3.1937 | 0.2025 |
| Lachnospira | 0.0044 | 0.0066 | 0.0057 | 0.0040 | 0.0068 | 0.0045 | 1.4960 | 0.4733 |
| Lachnospiraceae AC2044 group | 0.0000 | 0.0000 | 0.0044 | 0.0041 | 0.0017 | 0.0038 | 5.4073 | 0.0670 |
| Lachnospiraceae FCS020 group | 0.0018 | 0.0041 | 0.0004 | 0.0008 | 0.0019 | 0.0026 | 0.4673 | 0.7916 |
| Lachnospiraceae ND3007 group | 0.0018 | 0.0039 | 0.0045 | 0.0055 | 0.0025 | 0.0035 | 1.0478 | 0.5922 |
| Lachnospiraceae NK3A20 group | 0.0014 | 0.0032 | 0.0014 | 0.0027 | 0.0015 | 0.0034 | 0.0111 | 0.9945 |
| Lachnospiraceae NK4A136 group | 0.0034 | 0.0048 | 0.0074 | 0.0052 | 0.0081 | 0.0047 | 2.4924 | 0.2876 |
| Marvinbryantia | 0.0029 | 0.0026 | 0.0052 | 0.0013 | 0.0056 | 0.0034 | 3.6031 | 0.1650 |
| Oribacterium | 0.0021 | 0.0028 | 0.0024 | 0.0032 | 0.0012 | 0.0027 | 0.6287 | 0.7303 |
| Roseburia | 0.0012 | 0.0027 | 0.0000 | 0.0000 | 0.0065 | 0.0089 | 2.2902 | 0.3182 |
| Syntrophococcus | 0.0000 | 0.0000 | 0.0026 | 0.0051 | 0.0046 | 0.0074 | 2.2072 | 0.3317 |
| Lachnospiraceae uncultured | 0.0035 | 0.0055 | 0.0047 | 0.0029 | 0.0015 | 0.0011 | 2.6490 | 0.2659 |
| Peptococcus | 0.0009 | 0.0020 | 0.0008 | 0.0010 | 0.0021 | 0.0015 | 2.7173 | 0.2570 |
| [Eubacterium] coprostanoligenes group | 0.0288 | 0.0128 | 0.0262 | 0.0107 | 0.0295 | 0.0096 | 0.2029 | 0.9035 |
| Butyricicoccus | 0.0000 | 0.0000 | 0.0020 | 0.0029 | 0.0026 | 0.0020 | 5.3296 | 0.0696 |
| Candidatus Soleaferrea | 0.0005 | 0.0011 | 0.0023 | 0.0019 | 0.0024 | 0.0017 | 3.5170 | 0.1723 |
| Faecalibacterium | 0.0181 | 0.0111 | 0.0236 | 0.0162 | 0.0569 | 0.0259 | 6.9417 | 0.0311 |
| Fournierella | 0.0111 | 0.0073 | 0.0127 | 0.0069 | 0.0100 | 0.0080 | 0.3714 | 0.8305 |
| Intestinimonas | 0.0024 | 0.0023 | 0.0025 | 0.0029 | 0.0027 | 0.0037 | 0.0586 | 0.9711 |
| Negativibacillus | 0.0040 | 0.0016 | 0.0030 | 0.0022 | 0.0032 | 0.0009 | 0.2029 | 0.9035 |
| Oscillibacter | 0.0000 | 0.0000 | 0.0004 | 0.0007 | 0.0026 | 0.0041 | 2.4894 | 0.2880 |
| Oscillospira | 0.0074 | 0.0090 | 0.0105 | 0.0045 | 0.0063 | 0.0073 | 1.3146 | 0.5182 |
| Ruminiclostridium 5 | 0.0046 | 0.0033 | 0.0068 | 0.0036 | 0.0059 | 0.0017 | 1.8029 | 0.4060 |
| Ruminiclostridium 6 | 0.0017 | 0.0019 | 0.0020 | 0.0009 | 0.0002 | 0.0005 | 5.0979 | 0.0782 |
| Ruminiclostridium 9 | 0.0032 | 0.0048 | 0.0043 | 0.0035 | 0.0027 | 0.0028 | 0.4403 | 0.8024 |
| Ruminococcaceae NK4A214 group | 0.0239 | 0.0124 | 0.0274 | 0.0062 | 0.0331 | 0.0090 | 2.7857 | 0.2484 |
| Ruminococcaceae UCG-002 | 0.0174 | 0.0094 | 0.0206 | 0.0053 | 0.0206 | 0.0127 | 0.6000 | 0.7408 |
| Ruminococcaceae UCG-004 | 0.0009 | 0.0020 | 0.0023 | 0.0026 | 0.0013 | 0.0029 | 0.8203 | 0.6635 |
| Ruminococcaceae UCG-005 | 0.0464 | 0.0142 | 0.0591 | 0.0236 | 0.0439 | 0.0054 | 1.2857 | 0.5258 |
| Ruminococcaceae UCG-008 | 0.0077 | 0.0069 | 0.0065 | 0.0046 | 0.0039 | 0.0042 | 1.3115 | 0.5190 |
| Ruminococcaceae UCG-009 | 0.0025 | 0.0028 | 0.0023 | 0.0006 | 0.0017 | 0.0005 | 1.0714 | 0.5853 |
| Ruminococcaceae UCG-010 | 0.0027 | 0.0031 | 0.0025 | 0.0010 | 0.0031 | 0.0019 | 0.1117 | 0.9457 |
| Ruminococcaceae UCG-013 | 0.0009 | 0.0012 | 0.0003 | 0.0006 | 0.0002 | 0.0004 | 1.0591 | 0.5889 |
| Ruminococcaceae UCG-014 | 0.0331 | 0.0153 | 0.0311 | 0.0093 | 0.0351 | 0.0128 | 0.1800 | 0.9139 |
| Ruminococcus 1 | 0.0032 | 0.0028 | 0.0074 | 0.0052 | 0.0135 | 0.0081 | 5.5714 | 0.0617 |
| Ruminococcus 2 | 0.0051 | 0.0039 | 0.0076 | 0.0078 | 0.0157 | 0.0068 | 5.4527 | 0.0655 |
| Subdoligranulum | 0.0582 | 0.0427 | 0.0440 | 0.0075 | 0.0597 | 0.0111 | 3.7429 | 0.1539 |
| Ruminococcaceae uncultured | 0.0052 | 0.0058 | 0.0020 | 0.0018 | 0.0028 | 0.0031 | 1.0118 | 0.6030 |
| [Anaerorhabdus] furcosa group | 0.0004 | 0.0010 | 0.0011 | 0.0008 | 0.0005 | 0.0005 | 2.0820 | 0.3531 |
| Catenibacterium | 0.0051 | 0.0010 | 0.0081 | 0.0082 | 0.0170 | 0.0076 | 6.3514 | 0.0418 |
| Catenisphaera | 0.0089 | 0.0064 | 0.0071 | 0.0044 | 0.0041 | 0.0028 | 1.3000 | 0.5220 |
| Erysipelotrichaceae UCG-003 | 0.0003 | 0.0007 | 0.0003 | 0.0006 | 0.0004 | 0.0010 | 0.0111 | 0.9945 |
| Erysipelotrichaceae UCG-004 | 0.0005 | 0.0012 | 0.0005 | 0.0010 | 0.0003 | 0.0006 | 0.0830 | 0.9594 |
| Erysipelotrichaceae UCG-006 | 0.0009 | 0.0013 | 0.0013 | 0.0015 | 0.0013 | 0.0014 | 0.2541 | 0.8807 |
| Holdemanella | 0.0182 | 0.0127 | 0.0151 | 0.0075 | 0.0112 | 0.0070 | 1.0029 | 0.6057 |
| Sharpea | 0.0003 | 0.0007 | 0.0003 | 0.0007 | 0.0012 | 0.0027 | 0.0111 | 0.9945 |
| Solobacterium | 0.0133 | 0.0082 | 0.0060 | 0.0046 | 0.0094 | 0.0063 | 2.6914 | 0.2604 |
| Erysipelotrichaceae uncultured | 0.0123 | 0.0065 | 0.0195 | 0.0152 | 0.0050 | 0.0023 | 7.8600 | 0.0196 |
| Acidaminococcus | 0.0006 | 0.0014 | 0.0003 | 0.0007 | 0.0017 | 0.0029 | 0.7004 | 0.7045 |
| Phascolarctobacterium | 0.0056 | 0.0046 | 0.0101 | 0.0034 | 0.0094 | 0.0041 | 2.9314 | 0.2309 |
| Allisonella | 0.0007 | 0.0015 | 0.0000 | 0.0000 | 0.0006 | 0.0008 | 1.6706 | 0.4337 |
| Anaerovibrio | 0.0014 | 0.0020 | 0.0027 | 0.0025 | 0.0031 | 0.0047 | 0.6276 | 0.7307 |
| Dialister | 0.0008 | 0.0017 | 0.0017 | 0.0021 | 0.0046 | 0.0062 | 2.0253 | 0.3632 |
| Megasphaera | 0.0314 | 0.0291 | 0.0144 | 0.0150 | 0.0350 | 0.0237 | 1.9822 | 0.3712 |
| Mitsuokella | 0.0014 | 0.0018 | 0.0030 | 0.0043 | 0.0027 | 0.0047 | 0.0977 | 0.9523 |
| Schwartzia | 0.0005 | 0.0011 | 0.0000 | 0.0000 | 0.0004 | 0.0008 | 0.8769 | 0.6450 |
| Veillonellaceae uncultured | 0.0058 | 0.0130 | 0.0000 | 0.0000 | 0.0002 | 0.0000 | 0.8769 | 0.6450 |
| uncultured rumen bacterium | 0.0005 | 0.0012 | 0.0002 | 0.0005 | 0.0006 | 0.0013 | 0.0111 | 0.9945 |
| Succinivibrio | 0.0000 | 0.0000 | 0.0043 | 0.0042 | 0.0041 | 0.0071 | 5.1588 | 0.0758 |
| Escherichia-Shigella | 0.0007 | 0.0010 | 0.0003 | 0.0007 | 0.0004 | 0.0009 | 0.4673 | 0.7916 |
| Treponema 2 | 0.0010 | 0.0010 | 0.0028 | 0.0020 | 0.0013 | 0.0019 | 3.4793 | 0.1756 |
| Mollicutes RF39 uncultured bacterium | 0.0045 | 0.0030 | 0.0023 | 0.0013 | 0.0017 | 0.0014 | 1.3621 | 0.5061 |
| uncultured Lachnospiraceae bacterium | 0.0025 | 0.0045 | 0.0014 | 0.0017 | 0.0000 | 0.0000 | 2.8869 | 0.2361 |
| Unknown | 0.1118 | 0.0427 | 0.1425 | 0.0397 | 0.0888 | 0.0241 | 3.9514 | 0.1387 |
