# Supplementary material for: Effect of functional oils or probiotics on performance and microbiota profile of newly weaned piglets
Source: Sci Rep. 2021 Sep 30;11:19457. doi: 10.1038/s41598-021-98549-w (PMC8484476; doi:10.1038/s41598-021-98549-w)
Supplement: Supplementary file 5 — Supplementary Table S3. [file 41598_2021_98549_MOESM5_ESM.doc]

Table S3 - Ingredients and nutritional composition of experimental diets

| **Ingredients (%)** | Phase 1  28 to 43 days | Phase 2  43 to 57 days | Phase 3  57 to 66 days |
| --- | --- | --- | --- |
| Corn 7,86 % | 54.79 | 54.83 | 58.37 |
| Soybean meal 45 % | 22.47 | 29.25 | 27.58 |
| Pig nucleus^1^ | 10.00 | 10.00 | 0.00 |
| Pig nucleus II^2^ | 0.00 | 0.00 | 0.50 |
| Meat and bones meal 46 | 0.00 | 0.00 | 8.00 |
| Soybean oil | 2.90 | 2.91 | 3.65 |
| L-Lysine HCl | 0.02 | 0.03 | 0.40 |
| L-Threonine | 0.01 | 0.02 | 0.16 |
| DL-Methionine | 0.00 | 0.00 | 0.09 |
| Pig plasma | 5.00 | 0.50 | 0.00 |
| Sugar | 2.00 | 0.00 | 0.00 |
| Calcite limestone | 1.72 | 1.65 | 0.17 |
| Dicalcium phosphate | 0.38 | 0.00 | 0.00 |
| Salt | 0.00 | 0.11 | 0.38 |
| Mycotoxin adsorvent | 0.10 | 0.10 | 0.10 |
| Additive^3^ | 0.45 | 0.45 | 0.45 |
| Analyzed composition | | | |
| Metabolizable energy Kcal/kg | 3400 | 3375 | 3350 |
| Crude protein (%) | 19.5 | 19.35 | 21.25 |
| Lysine Dig. (%) | 1.45 | 1.35 | 1.25 |
| Methionine Dig. (%) | 0.45 | 0.27 | 0.36 |
| Threonine Dig. (%) | 0.97 | 0.90 | 0.81 |
| Tryptophan Dig. (%) | 0.29 | 0.23 | 0.22 |
| Calcium (%) | 1.07 | 0.97 | 1.10 |
| Phosphorus Dig. (%) | 0.37 | 0.30 | 0.57 |
| Sodium (%) | 0.20 | 0.22 | 0.23 |
| Chlorine (%) | 0.13 | 0.20 | 0.37 |
| Potassium (%) | 0.62 | 0.71 | 0.74 |
| Crude fiber (%) | 2.24 | 2.60 | 2.47 |

^1^ Pig nucleus - guarantee levels: humidity (max) 130g/kg; folic acid (min.) 14mg/kg; pantothenic acid 295mg/kg; biotin 1.0mg/kg; kg; calcium (min-max) 25 - 45g; cobalt (min.) 4.5mg/kg; copper (min.) 1200mg/kg; choline (min.) 4500mg/kg; chlorine (min) 95g/kg; chromium (min.) 2mg/kg; ether extract (min.) 1000 mg/kg; iron (min.) 800mg/kg; crude fiber (max.) 10g/kg; phytase 5000FTU/ kg; phosphorus (min.) 20g/kg; iodine (min.) 13mg/kg; lysine (min) 45g/kg; manganese (min.) 650mg/kg; mineral matter (max.) 300g/kg; methionine (mín.) 21g/kg; niacina (min.) 300mg/kg; crude protein (min.) 40g/kg; sodium (min.) 15g/kg; selenium (min.) 4mg/kg; threonine (min.) 28g/kg; tryptophan (min.) 5600mg/kg; vitamin A (min.) 120000 IU/kg; vitamin B1 (min.) 18mg/kg; vitamin B12 (min) 280mcg/kg; vitamin B2 (min) 50mg/kg; vitamin B6 (min.) 50mg/kg; vitamin D3 (min.) 19000 IU/kg; vitamin E (min.) 850 IU/kg; zinc (min.) 18.5g/kg. ^2^ Pig nucleus II - guarantee levels: folic acid (min.) 290mg/kg; pantothenic acid 5900mg/kg; beta glucanase 5000mg/kg; biotin 19mg/kg; cobalt (min.) 90mg/kg; copper (min.) 23g/kg; choline (min.) 95g/kg; iron (min.) 16g/kg; phytase 1000FTU/ kg; iodine (min.) 260mg/kg; manganese (min.) 13g/kg; niacina (min.) 5800mg/kg; selenium (min.) 85mg/kg; vitamin A (min.) 2400,000 IU/kg; vitamin B1 (min.) 390mg/kg; vitamin B12 (min) 5900mcg/kg; vitamin B2 (min) 1000mg/kg; vitamin B6 (min.) 950mg/kg; vitamin D3 (min.) 380000 IU/kg; vitamin E (min.) 17000 IU/kg; vitamin K3 (min) 480 mg/kg; Xylanase 5000 mg/kg; zinc (min.) 28g/kg. ^3^Additives according to treatments. Control: without additives; Blend: Oils: 0.15% Integrity_Oligo + 0.20% Essential_Oligo; Probiotics: 0.60%.
